# Genome-Wide Scan on Total Serum IgE Levels Identifies *FCER1A* as Novel Susceptibility Locus

**DOI:** 10.1371/journal.pgen.1000166

**Published:** 2008-08-22

**Authors:** Stephan Weidinger, Christian Gieger, Elke Rodriguez, Hansjörg Baurecht, Martin Mempel, Norman Klopp, Henning Gohlke, Stefan Wagenpfeil, Markus Ollert, Johannes Ring, Heidrun Behrendt, Joachim Heinrich, Natalija Novak, Thomas Bieber, Ursula Krämer, Dietrich Berdel, Andrea von Berg, Carl Peter Bauer, Olf Herbarth, Sibylle Koletzko, Holger Prokisch, Divya Mehta, Thomas Meitinger, Martin Depner, Erika von Mutius, Liming Liang, Miriam Moffatt, William Cookson, Michael Kabesch, H.-Erich Wichmann, Thomas Illig

**Affiliations:** 1 Department of Dermatology and Allergy, Technische Universität München, München, Germany; 2 Division of Environmental Dermatology and Allergy, Helmholtz Zentrum München, Neuherberg and ZAUM-Center for Allergy and Environment, Technische Universität München, München, Germany; 3 Institute of Epidemiology, Helmholtz Zentrum München, German Research Center for Environmental Health, Neuherberg, Germany; 4 Institute of Medical Informatics, Biometry and Epidemiology, Ludwig-Maximilians-Universität München, München, Germany; 5 IMSE Institute for Medical Statistics and Epidemiology, Technische Universität München, München, Germany; 6 Graduate School of Information Science in Health (GSISH), Technische Universität München, München, Germany; 7 Department of Dermatology and Allergy, University of Bonn, Bonn, Germany; 8 IUF–Institut für Umweltmedizinische Forschung at the Heinrich-Heine-University, Düsseldorf, Germany; 9 Marien-Hospital, Wesel, Germany; 10 Department of Pediatrics, Technische Universität München, München, Germany; 11 Department of Human Exposure Research and Epidemiology, UFZ–Centre for Environmental Research Leipzig, Leipzig, Germany; 12 University Children's Hospital, Ludwig-Maximilians-Universität München, München, Germany; 13 Institute of Human Genetics, Helmholtz Zentrum München, German Research Center for Environmental Health, Neuherberg, Germany; 14 Institute of Human Genetics, Klinikum rechts der Isar, Technische Universität München, München, Germany; 15 Center for Statistical Genetics, Department of Biostatistics, School of Public Health, Ann Arbor, Michigan, United States of America; 16 National Heart and Lung Institute, Imperial College London, London, United Kingdom; University of Pennsylvania, United States of America

## Abstract

High levels of serum IgE are considered markers of parasite and helminth exposure. In addition, they are associated with allergic disorders, play a key role in anti-tumoral defence, and are crucial mediators of autoimmune diseases. Total IgE is a strongly heritable trait. In a genome-wide association study (GWAS), we tested 353,569 SNPs for association with serum IgE levels in 1,530 individuals from the population-based KORA S3/F3 study. Replication was performed in four independent population-based study samples (total n = 9,769 individuals). Functional variants in the gene encoding the alpha chain of the high affinity receptor for IgE (*FCER1A*) on chromosome 1q23 (rs2251746 and rs2427837) were strongly associated with total IgE levels in all cohorts with *P* values of 1.85×10^−20^ and 7.08×10^−19^ in a combined analysis, and in a post-hoc analysis showed additional associations with allergic sensitization (*P* = 7.78×10^−4^ and *P* = 1.95×10^−3^). The “top” SNP significantly influenced the cell surface expression of FCER1A on basophils, and genome-wide expression profiles indicated an interesting novel regulatory mechanism of FCER1A expression via GATA-2. Polymorphisms within the *RAD50* gene on chromosome 5q31 were consistently associated with IgE levels (*P* values 6.28×10^−7^−4.46×10^−8^) and increased the risk for atopic eczema and asthma. Furthermore, *STAT6* was confirmed as susceptibility locus modulating IgE levels. In this first GWAS on total IgE *FCER1A* was identified and replicated as new susceptibility locus at which common genetic variation influences serum IgE levels. In addition, variants within the *RAD50* gene might represent additional factors within cytokine gene cluster on chromosome 5q31, emphasizing the need for further investigations in this intriguing region. Our data furthermore confirm association of *STAT6* variation with serum IgE levels.

## Author Summary

High levels of serum IgE are considered markers of parasite and helminth exposure. In addition, they are associated with allergic disorders, play a key role in anti-tumoral defence, and are crucial mediators of autoimmune diseases. There is strong evidence that the regulation of serum IgE levels is under a strong genetic control. However, despite numerous loci and candidate genes linked and associated with atopy-related traits, very few have been associated consistently with total IgE. This study describes the first large-scale, genome-wide scan on total IgE. By examining >11,000 German individuals from four independent population-based cohorts, we show that functional variants in the gene encoding the alpha chain of the high affinity receptor for IgE (*FCER1A*) on chromosome 1q23 are strongly associated with total IgE levels. In addition, our data confirm association of *STAT6* variation with serum IgE levels, and suggest that variants within the *RAD50* gene might represent additional factors within cytokine gene cluster on chromosome 5q31, emphasizing the need for further investigations in this intriguing region.

## Introduction

High levels of IgE have been considered for many years as markers of parasite and helminth exposure to which they confer resistance [1]. In Western lifestyle countries with less contact, however, elevated IgE levels are associated with allergic disorders [2]. Only recently, it has been established that IgE antibodies also play a key role in anti-tumoral defence [3] and are crucial mediators of autoimmune diseases [4], thus challenging the traditional Th1/Th2 dogma.

High total serum IgE levels are closely correlated with the clinical expression and severity of asthma and allergy [5],[6]. The regulation of serum IgE production is largely influenced by familial determinants, and both pedigree- and twin-based studies provided evidence of a strong genetic contribution to the variability of total IgE levels [7],[8]. Genetic susceptibility of IgE-responsiveness is likely to be caused by a pattern of polymorphisms in multiple genes regulating immunologic responses[9], but so far only very few loci could be established consistently and robustly, most notable *FCER1B*, *IL-13* and *STAT6* [10],[11].

Family and case-control studies indicated that total serum IgE levels are largely determined by genetic factors that are independent of specific IgE responses and that total serum IgE levels are under stronger genetic control than atopic disease [8],[12],[13],[14]. An understanding of the genetic mechanisms regulating total serum IgE levels might also aid in the dissection of the genetic basis of atopic diseases. In an attempt to identify novel genetic variants that affect total IgE levels, we conducted a genome-wide association study (GWAS) in 1,530 German adults and replicated the top signals in altogether 9,769 samples of four independent study populations.

## Results

### Genome-wide Association Scan

**Figure 1:**
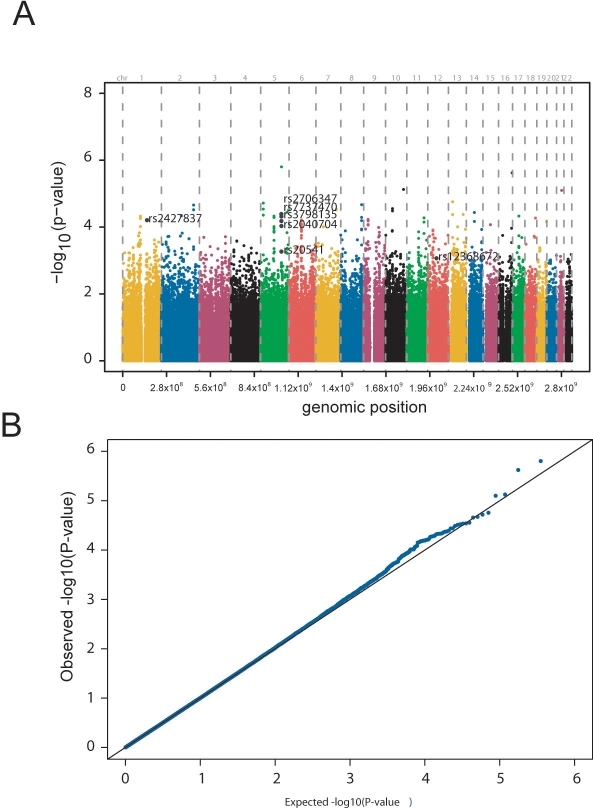
Results of the KORA S3/F3 500 K analysis. a) Genome-wide association study of chromosomal loci for IgE levels: the analysis is based on a population-based sample of 1530 persons. The x-axis represents the genomic position of 353,569 SNPs, and the y-axis shows −log10 (*P* value). b) Quantile-quantile plot of *P* values: Each black dot represents an observed statistic (defined as the −log10( *P* value)) versus the corresponding expected statistic. The line corresponds to the null distribution.

For the GWAS 1,530 individuals from the population-based KORA S3/F3 500 K study with available total IgE levels were typed with the Affymetrix 500 K Array Set. For statistical analysis, we selected SNPs by including only high-quality genotypes to reduce the number of false positive signals. A total of 353,569 SNPs passed all quality control measures and were tested for associations with IgE levels. Figure 1 summarizes the results of the KORA S3/F3 500 K analysis. No single SNPs reached genome-wide significance, but the scan pointed to the gene encoding the alpha chain of the high affinity receptor for IgE (*FCER1A*) on chromosome 1 (Figure 1A). Particularly the quantile-quantile-plot of the *P* values illustrates observed significant associations beyond those expected by chance (Figure 1B).

### Replication and Fine-Mapping

For replication in the independent population-based KORA S4 cohort (N = 3,890), we used the following inclusion criteria: (i) *P*<10^−4^ in the genome wide analysis (39 SNPs, 35 expected); (ii) *P*<10^−3^ with at least one neighboring SNPs (±100 kb) with *P*<10^−3^ (45 SNPs). The specific results for all SNPs in the GWAS and KORA S4 are given in supplementary table S3. Six SNPs were significantly associated with total IgE levels in KORA S4 with *P* values ranging from 2.47×10^−4^ to 3.23×10^−9^ (given a Bonferroni-corrected significance level of 5.10×10^−4^). The strongest associations were observed for rs2427837 (*P* = 3.23×10^−9^), which is located in the 5′ region of *FCER1A*, and rs12368672 (*P* = 2.03×10^−6^), which is located in the 5′ region of *STAT6*. In addition, all 4 *RAD50* SNPs which had been selected in the GWAS could be replicated.

**Figure 2:**
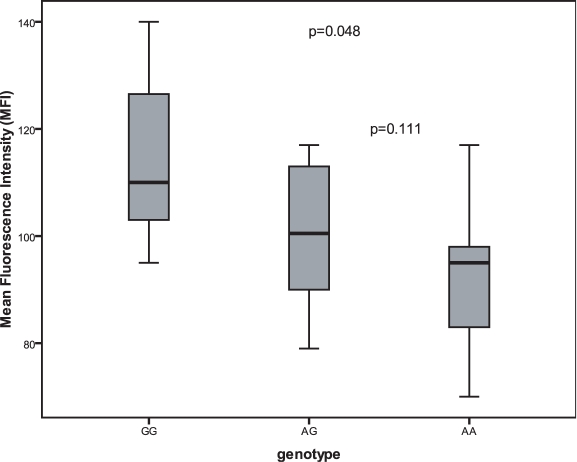
Expression of the FCER1 alpha chain on IgE-stripped basophils. PBMCs were isolated from individuals displaying high sIgE levels and FCER1 alpha chain expression was measured after stripping IgE from its receptor by lactic acid buffer incubation by FACS. Results are expressed as mean fluorescence intensity for FCER1A in the basophile gate. Significance was calculated using the Student's-t-test.

Effect estimates of the SNPs in *FCER1A* and *STAT6* were only slightly lower compared to those in the KORA S3/F3 500 K sample whereas clearly lower effects were observed for the SNPs in *RAD50*. The rare allele “G” of the top ranked SNP rs2427837 in *FCER1A* had an estimated effect per copy of −0.212 based on the logarithm of total IgE. This translates into an estimated decrease of 19.1% in total serum IgE level for the heterozygote genotype and 34.6% for the rare homozygote genotype, which was significantly associated with an increased FCER1A expression on IgE-stripped basophils (Figure 2).

The estimated effect of the *STAT6* SNP rs12368672 was 0.156 resulting in an increase of total IgE of 16.9% and 36.6% for the heterozygote and rare homozygote genotype, respectively. The most significant SNP in the *RAD50* gene (rs2706347) had an effect estimate of 0.143 (*P* = 2.26×10^−4^) with an associated increase in total IgE of 15.4% and 33.1%. Altogether the variance of total IgE level explained by genotypes of the three replicated regions was about 1.9%.

**Figure 3:**
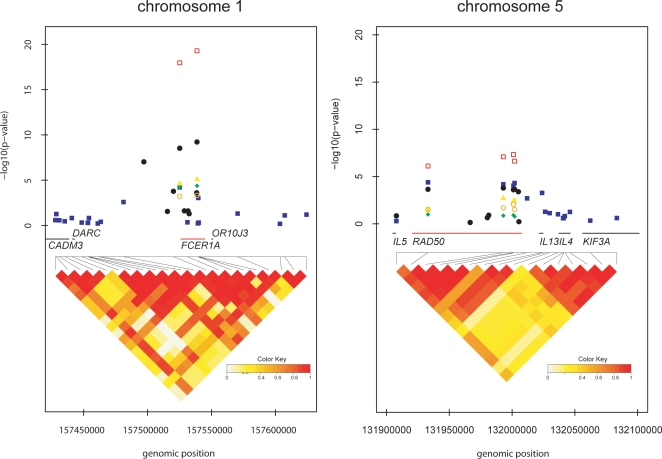
P value and pairwise linkage disequilibrium diagram of the region on chromosome 1q23, area of *FCER1A* (panel A), and chromosome 5q31, area of *RAD50* (panel B). Pairwise LD, measured as D', was calculated from KORA S3/F3 500 K. Shading represents the magnitude of pairwise LD with a white to red gradient reflecting lower to higher D' values. Gene regions are indicated by colored bars. *P* value diagram: The x-axis represents the genomic position. The y-axis shows −log10 (*P* values) of KORA S3/F3 500 K (blue), KORA S4 (black), GINI (yellow), LISA (green), ISAAC (orange), combined replication samples (red).

To fine-map the regions of strong association in greater detail, we selected additional SNPs covering the *FCER1A* and *RAD50* gene region based on HapMap data from individuals of European ancestry. In addition, two previously described promoter SNPs of *FCER1A* (rs2251746, rs2427827) [15],[16], as well as 2 SNPs in the *RAD50* hypersensitive site 7 (RHS7) in intron 24 (rs2240032, rs2214370)[17] were included. In total, 14 SNPs were genotyped in KORA S4. We found the strongest association in the proximal promoter region of the *FCER1A* gene, at rs2251746, which was in strong LD (r^2^ = 0.96) with rs2427837 (Table 1 and Figure 3). The contribution of the two alleles of rs2251746 in homozygotes and heterozygotes is given in Figure S1. Their effect is observed across the full range of IgE values. The strongest observed association of SNP rs2251746 and the distribution of the SNPs in the region are shown in Figure 3A. None of the *RAD50* SNPs in the fine-mapping showed distinctly stronger association with total IgE (Figure 3B). We additionally sequenced all *FCER1A* exons with adjacent intronic sequences in 48 male and 48 female samples selected equally from the extremes of the serum IgE distribution in 3,890 individuals from the KORA S4 cohort. We identified two new mutations, each present in one individual only, and concurrently confirmed three SNPs already annotated in public databases (dbSNP) with validated minor allele frequencies in Europeans. None of the novel mutations were predicted to have functional consequences (for details see Text S1 and Tables S5 and S6). Haplotype analysis for the *FCER1A* gene showed lower total IgE levels with effect estimates ranging from −0.18 to −0.32 for a haplotype described by the rare “G” allele of rs2427837 and the rare “C” allele of rs2251746 (haplotype frequency 26.4%) in comparison to all other common haplotypes carrying both major alleles (Table S7).

**Table 1:** Association between total IgE and selected SNPs in the GWAS sample and in the four replication samples.

		GWAS KORA S3/F3			Replication KORA S4			Replication GINI			Replication LISA			Replication ISAAC			Combined		
		n = 1,530			n = 3,890			n = 1,839			n = 1,042			n = 2,998			n = 9,769		
Gene	SNP	Est.	P value	Est. %	Est.	P value	Est. %	Est.	P value	Est. %	Est.	P value	Est. %	Est.	P value	Est. %	Est.	P value	Est. %
FCER1A	rs2511211				−0.206	9.28E-08	−18.59												
FCER1A	rs10489854				0.153	2.85E-02	16.52												
FCER1A	rs2494262				0.122	1.67E-04	12.99												
FCER1A	rs2427837	−0.235	6.19E-05	−20.94	−0.212	3.23E-09	−19.12	−0.219	2.51E-05	−19.64	−0.280	6.58E-05	−24.56	−0.145	4.27E-04	−13.53	−0.202	7.08E-19	−18.27
FCER1A	rs12565775				0.119	2.45E-02	12.56												
FCER1A	rs2427824				0.082	2.52E-02	8.49												
FCER1A	rs3845625				0.085	5.09E-02	8.82												
FCER1A	rs2427827				0.120	2.45E-04	12.72												
FCER1A	rs2251746				−0.227	6.07E-10	−20.29	−0.236	8.14E-06	−20.99	−0.290	4.18E-05	−25.17	−0.153	2.11E-04	−14.16	−0.213	1.85E-20	−19.21
RAD50	rs2069812				−0.052	1.42E-01	−4.98												
RAD50	rs2706347	0.236	4.05E-05	26.62	0.143	2.26E-04	15.43	0.122	2.91E-02	13.02	0.118	1.01E-01	12.56	0.095	2.70E-02	9.96	0.120	6.28E-07	12.80
RAD50	rs6884762				0.034	7.22E-01	3.46												
RAD50	rs17772565				−0.096	2.27E-01	−9.17												
RAD50	rs17772583				−0.058	1.24E-01	−5.62												
RAD50	rs3798135	0.227	6.58E-05	25.48	0.142	2.32E-04	15.20	0.173	2.00E-03	18.91	0.107	1.37E-01	11.26	0.101	1.75E-02	10.64	0.129	6.69E-08	13.82
RAD50	rs2040704	0.221	9.25E-05	24.73	0.140	2.47E-04	14.97	0.158	4.40E-03	17.14	0.111	1.21E-01	11.73	0.112	8.22E-03	11.83	0.130	4.46E-08	13.90
RAD50	rs7737470	0.231	4.81E-05	25.99	0.142	2.27E-04	15.28	0.163	3.70E-03	17.70	0.100	1.64E-01	10.55	0.087	4.13E-02	9.12	0.123	3.35E-07	13.07
RAD50	rs2240032				0.137	4.01E-04	14.67												
RAD50	rs2214370				0.136	5.95E-01	14.54												
STAT6	rs12368672	0.167	8.52E-04	18.18	0.156	2.03E-06	16.93	0.016	7.34E-01	1.65	0.075	2.44E-01	7.78				0.108	1.52E-05	11.44

For further replication of the KORA S4 results in the population-based children cohorts GINI (n = 1,839), LISA (n = 1,042) and ISAAC (n = 2,998) the top 6 SNPs: rs2251746, rs2427837, rs2040704, rs2706347, rs3798135, rs7737470 and rs12368672 were tested for association with total serum IgE levels. In GINI, all SNPs except rs12368672 yielded significant *P* values ranging from 0.029 to 8.14×10^−6^. After correction for multiple testing SNP rs2706347 is slightly above the significance level. In LISA, the two *FCER1A* polymorphisms rs2251746 and rs2427837 were strongly associated (*P* = 4.18×10^−5^ and 6.58×10^−5^), while the *RAD50* SNPs showed consistent trends, but no statistical significance. In ISAAC, the effect estimates of the two *FCER1A* SNPs were distinctly smaller than in the other replication samples but in the same direction and significantly associated with *P* values of 2.11×10^−4^ for rs2251746 and of 4.27×10^−4^ for rs2427837. The *RAD50* SNPs showed effect estimates in concordance with the other replication samples but were only borderline significant. Additional analysis of markers in the *RAD50-IL13* region in a subset of 526 children from the ISAAC replication cohort (for details see Table S9) indicated presence of one linkage disequilibrium (LD) block, which encompasses the entire *RAD50* gene and extends into the promoter region of the *IL13* gene, whereas rs20541 showed low levels of LD with *RAD50* variants (r2<0.3) (Figure S2)

In the combined analysis of all replication samples both selected *FCER1A* SNPs (*P* = 1.85×10^−20^ and 7.08×10^−19^ for rs2251746 and rs2427837, respectively) and *RAD50* SNPs (*P* = 6.28×10^−7^−4.46×10^−8^) were significantly associated with IgE levels. Effect estimates were consistent throughout all replication cohorts.

### Association Analysis with Dichotomous Traits

In a *post hoc* analysis of the KORA S4 and ISAAC replication cohorts, *FCER1A* polymorphisms rs2251746 and rs2427837 showed association with allergic sensitization (*P* = 7.78×10^−4^ and 1.95×10^−3^ in KORA, *P* = 0.025 and 0.032 in ISAAC), while there were no significant associations for the dichotomous traits asthma, rhinitis and atopic eczema (AE). However, the number of cases for these traits was relatively low. We therefore additionally typed a cohort of 562 parent-offspring trios for AE from Germany and a population of 638 asthma cases and 633 controls from UK. In these cohorts we observed weak associations of *RAD50* variants with eczema (*P* = 0.007–0.01) and with asthma (*P* = 0.017–0.002, Table S8).

## Discussion

In this large-scale population-based GWAS with follow-up investigations in 9,769 individuals from 4 independent population-based study samples we show that functional variants of the gene encoding the alpha chain of the high affinity receptor for IgE (*FCER1A*) are of major importance for the regulation of IgE levels.

The high affinity receptor for IgE represents the central receptor of IgE-induced type I hypersensitivity reactions such as the liberation of vasoactive mediators including serotonin and histamine, but also for the induction of profound immune responses through the activation of NFkappa B and downstream genes [18]. It is usually expressed as a αβγ_2_ complex on mast cells and basophils, but additionally as a αγ_2_ complex on antigen-presenting cells (APCs) as shown for dendritic cells and monocytes [18]. Interestingly, in APCs, IgE-recognition of allergens also leads to facilitated allergen uptake via FCER1 and thereby contributes to a preferential activation of Th2-subsets of T-cells. Its expression is substantially influenced by the binding of IgE to either form of the receptor as bound IgE apparently protects the receptor from degradation and thus enhances surface expression without *de novo* protein synthesis. Of note, binding of IgE in the two different complexes only uses the alpha subunit of the receptor lacking contact sites with the beta or gamma subunits. Consequently, the expression level of the alpha subunit is crucial for IgE levels on immune cells [18].

Previous studies suggested linkage of atopy to the gene encoding the β chain of the high-affinity IgE receptor (*FCRER1B*) [19]. FCER1B plays a critical role in regulating the cellular response to IgE and antigen through its capacity to amplify FCER1 signalling and regulate cell-surface expression [18], and there have been several studies which reported an association of *FCER1B* variants and atopy-related traits but conflicting results for total IgE [20],[21],[22],[23],[24],[25],[26],[27],[28]. In a more recent study, no association between *FCER1B* tagSNPs and IgE levels was observed [22]. The 500 k random SNP array contained only one SNP within as well as 31 SNPs within a 100-kb region around this gene, which were not significantly associated with total IgE. However, we cannot rule out that we missed relevant variants in this gene.

In the present study we identified *FCER1A* as susceptibility locus in a genome-wide association scan and replicated association of the *FCER1A* polymorphism rs2427837 with serum IgE levels in a total of 9,769 individuals from 4 independent population-based cohorts with a combined *P* value of 7.08×10^−19^. This SNP is in complete LD with the *FCER1A* polymorphism rs2251746, for which we observed a combined *P* value of 1.85×10^−20^.

Besides the continuous cycling of the IgE receptor subunits from intracellular storage pools to the surface, there is also a substantial expression of the alpha subunit after stimulation with IL-4 which requires *de novo* protein synthesis [18]. This induction is stimulated by the transcription factor GATA-1, which has a binding site in the putative promoter region of the *FCER1A* gene. Notably, in a previous study with Japanese individuals it could be shown that the minor allele of the polymorphism rs2251746 is associated with higher FCER1A expression through enhanced GATA-1 binding [15]. In line with this we observed an increased cell surface expression of FCER1A on IgE-stripped basophils from individuals homozygous for the “G” allele at rs2427837 (Figure 2). Analysis of the correlation of FCER1A expression with IgE levels in 320 KORA samples where whole genome blood expression profiles were available revealed no significant effect. However, FCER1A expression showed a significant dependency on IL-4 (*P* = 0.0087) and GATA-1 expression (*P* = 1.4×10^−4^), confirming the known stimulation pathway. Interestingly, we found a highly significant dependency of FCER1A expression on GATA-2 transcript levels (p = 7.8×10^−27^). While whole blood expression levels could easily obscure the situation in basophils, this finding might indicate a novel regulatory mechanisms of FCER1A expression via GATA-2 [18].

The large (>50 kb) *RAD50* gene, which encodes an ubiquitously expressed DNA repair protein, is located within the Th2-cytokine locus on chromosome 5q31, which has been linked with total IgE [29]. It contains multiple conserved non-coding sequences with presumed regulatory function [30]. Remarkably, evidence has been provided for the presence of a locus control region (LCR) within a 25 kb segment of the 3′ region of this gene, which plays an important role in the regulation of Th2 cytokine gene transcription [31]. The core of this LCR is constituted by four *RAD50* hypersensitive sites (RHS) in intron 21 (RHS4-6) and 24 (RHS7) [17],[32],[33]. The finding of an association between *RAD50* variants and IgE levels is new and biologically compelling. However, it has to be considered that so far *RAD50* has not emerged as candidate, but that several known candidate genes for atopy-related traits map to this region with strong linkage disequlibrium, especially *IL13*, which is one of the strongest and widely replicated candidate genes [10],[11]. Notably, two functional *IL13* polymorphisms, *IL1*3-1112CT (rs1800925) in the promoter region and *IL13*+2044GA (*IL13* Arg130Gln, rs20541) in Exon 4, have been shown to be associated with a range of atopy-related disorders. *IL13*+2044GA (rs20541) did not pass our selection criteria, and *IL1*3-1112CT (rs1800925) is not contained in the Affymetrix 500 K Array Set. Additional analysis of markers in this region including these two SNPs showed one LD block encompassing the entire *RAD50* gene and extending into the *IL13* promoter region, whereas rs20541 showed low levels of LD with *RAD50* SNPs (Figure S2). Thus, we cannot reliably differentiate the specific source of the signal between *RAD50* and *IL13* in our data. Functional studies are needed to assess whether *RAD50* is a true causal gene and to identify the causal genetic variants modulating IgE levels in this region.

The identification and positive replication of the *STAT6* locus, which is located in one of the most frequently identified genomic regions linked to atopy-related phenotypes [34], serves as positive control for the experiment. Our results confirm previous candidate studies which showed that genetic variants in the gene encoding STAT6, a key regulatory element of the TH2 immune response, contribute to the regulation of total serum IgE [35],[36].

Other previously reported candidate genes for total IgE showed no or only weak signals in our genome-wide scan (Tables S10 and S11). However, it has to be considered that there are only very few genes that have been associated in the first place to IgE such as *STAT6*, whereas most reported candidate genes for total IgE were investigated in asthma or eczema cohorts [10],[11]. In addition, there have been queries with regard to replication for many of the genes reported. Thus, our data obtained in a population-based and ethnically homogeneous sample (South German Caucasians) are not readily comparable with previous candidate gene studies. Furthermore some previously implicated variants were covered insufficiently by the 500 k random SNP array (Table S10).

In summary, in this first GWAS on total IgE *FCER1A* was identified and replicated as new susceptibility locus at which common genetic variation influences serum IgE levels. In addition, our data suggest that variants within the *RAD50* gene might represent additional factors within cytokine gene cluster on chromosome 5q31, emphasizing the need for further investigations in this intriguing region.

## Methods

### Subjects and Study Design

A detailed description of the GWAS population and the replication samples is given in Text S1 and Table S1. In all studies informed consent has been given, and all studies have been approved by the local ethical committees. The participants were of European origin.

### KORA S3/F3 500 K and Replication Sample KORA S4

The study population for the GWAS (KORA S3/F3 500 K) and the first replication cohort were recruited from the KORA S3 and S4 surveys. Both are independent population-based samples from the general population living in the region of Augsburg, Southern Germany, and were examined in 1994/95 (KORA S3) and 1999/2001 (KORA S4). The standardized examinations applied in both surveys have been described in detail elsewhere [37]. In the KORA S3 study 4,856 subjects (participation rate 75%), and in KORA S4 in total 4,261 subjects have been examined (participation rate 67%). 3,006 subjects participated in a follow-up examination of S3 in 2004/05 (KORA F3). For KORA S3/F3 500 K we selected 1,644 subjects of these participants in the age range 25 to 69 years including 1,530 individuals with total IgE level available. From KORA S4, DNA samples from 3,890 individuals with total IgE level were available. Total and specific IgE antibodies to aeroallergens (S×1) were measured using RAST FEIA CAP system (Pharmacia, Freiburg, Germany). Specific sensitization was defined as specific IgE levels ≥0.35KU/l (CAP class > = 1).

### GINI and LISA Replication Samples

GINI (German Infant Nutritional Intervention Program) and LISA (Influences of lifestyle-related factors on the immune system and the development of allergies in childhood study) are two ongoing population-based birth cohorts conducted in Germany. A detailed description of screening and recruitment has been provided elsewhere [38]. Briefly, the GINI birth cohort comprises 5,991 newborns, who were recruited between January 1996 and June 1998 in 16 maternity wards in Wesel and Munich, Germany. Children with a positive medical history of atopic disease were invited to a randomized clinical trial with hydrolyzed formulae [39]. The LISA birth cohort study includes 3,097 neonates who were recruited between December 1997 and January 1999 in Munich, Leipzig and Wesel, Germany. Blood samples were collected from 1,962 (51%) and 1,193(50%) children from the GINI and LISA study, respectively, at age 6. Total IgE was determined by standardized methods with CAP-RAST FEIA (Pharmacia Diagnostics, Freiburg, Germany).

### ISAAC Replication Sample

Between 1995 and 1996, a cross sectional study was performed in Munich and in Dresden, Germany as part of the International Study of Asthma and Allergy in Childhood phase II (ISAAC II) to assess the prevalence of asthma and allergies in all schoolchildren attending 4^th^ class in both cities (age 9 to 11 years) [40]. Serum measurements for total and specific IgE were performed according to standardized procedures as previously described [40]. Allergic sensitization was defined as positive prick test reaction to at least one out of six common aeroallergens. Within the study population of 5,629 children, all children of German origin with DNA and total IgE level available were included in this analysis (n = 2,998).

### KORA S3/F3 500 K Genotyping and Quality Control

Genotyping for KORA S3/F3 500 K was performed using Affymetrix Gene Chip Human Mapping 500 K Array Set consisting of two chips (Sty I and Nsp I). Genomic DNA was hybridized in accordance with the manufacturer's standard recommendations. Genotypes were determined using BRLMM clustering algorithm. We performed filtering of both conspicuous individuals and single nucleotide polymorphisms (SNPs) to ensure robustness of association analysis. Details on quality criteria are described in Text S1 and Table S2.

### SNP Selection for Replication and Fine-Mapping

The power of the replication was estimated for a difference in log total IgE per allele of 0.2 and a nominal significance level of 0.05. The power to detect a true association was above 85% in KORA S4, GINI and ISAAC; whereas in LISA it was about 55%. No single SNPs in the GWAS reached genome-wide significance using a Bonferroni threshold of 1.4×10^−7^. To fine map the replicated loci in KORA S4 we selected tagging SNPs and used the pairwise tagging algorithm (r^2^>0.8) implemented in HAPLOVIEW 3.3 (HapMap data release #22, March 2007, on NCBI B36 assembly, dbSNP b126) and additionally selected putative functional SNPs in *FCER1A* and *RAD50*.

### SNP Genotyping and Quality Control in the Replication Samples

In all replication samples genotyping of SNPs was realized with the iPLEX (Sequenom San Diego, CA, USA) method by means of matrix assisted laser desorption ionisation-time of flight mass spectrometry method (MALDI-TOF MS, Mass Arraay, Sequenom, San Diego, CA, USA) according to the manufacturers instructions. In KORA S4 for 7 of 84 replicated SNPs a deviation from Hardy-Weinberg-Equilibrium was observed (*P* value<0.01). In LISA, GINI and ISAAC all replicated SNPs were in HWE. Details on genotyping are described in Text S1 and Table S4.

### Mutational Analysis by Cycle Sequencing

*FCER1A* exons were amplified with intronic primers (Tables S5 and S6) and were directly sequenced using a BigDye Cycle sequencing kit (Applied Biosystems). Genomic DNA (∼30 ng) was subjected to PCR amplification carried out in a 15 µl volume containing 1× PCR Master Mix (Promega), 0.25 µM of each forward and reverse primer under the following cycle conditions: initial step at 95°C for 5 min, for 30 cycles at 95°C for 30 s, 58°C (exon 1 62°C) for 30 s, and 72°C for 30 s; and final extension at 72°C for 5 min.

### Statistical Analysis of Genetic Effects

In the KORA S3/F3 500 K sample possible population sub-structures were analyzed (Text S1). Additive genetic models assuming a trend per copy of the minor allele were used to specify the dependency of logarithmic values of total IgE levels on genotype categories. The result is a multiplicative model on the original scale of total IgE with effects interpreted in percental changes. All models were adjusted for gender and in the adult cohorts we adjusted additionally for age. We used a linear regression algorithm implemented in the statistical analysis system R (http://www.r-project.org/) and SAS (Version 9.1.). To select significant SNPs in the genome-wide screening and the replications we used conservative Bonferroni thresholds which corresponded to a nominal level of 0.05. Haplotype reconstruction and haplotype association analysis was performed in the KORA S4 replication sample using the R-library *HaploStats* that allows including all common haplotypes in the linear regression and incorporating age and gender as covariates. The most common haplotype served as reference. Details on haplotype analysis are described in Text S1.

### Gene Expression Analysis

Peripheral blood (2.5 ml) was drawn from individuals participating in the KORA study under fasting conditions. The blood samples were collected between 10–12am directly in PAXgene (TM) Blood RNA tubes (PreAnalytiX). The RNA extraction was performed using the PAXgene Blood RNA Kit (Qiagen). RNA and cRNA quality control was carried out using the Bioanalyzer (Agilent) and quantification was done using Ribogreen (Invitrogen). 300–500 ng of RNA was reverse transcribed into cRNA and biotin-UTP labeled using the Illumina TotalPrep RNA Amplification Kit (Ambion). 1,500 ng of cRNA was hybridized to the Illumina Human-6 v2 Expression BeadChip. Washing steps were carried out in accordance with the Illumina technical note # 11226030 Rev. B. The raw data were exported from the Illumina “Beadstudio” Software to R. The data were converted into logarithmic scores and normalized using the LOWESS method [41]. The association between *FCER1A* gene expression (independent variable) and IgE level (dependent variable) was computed using the linear regression model adjusted for gender.

## Supporting Information

Figure S1 Box plot comparing the total IgE levels for the genotypes at rs2251746. The x axis represents the three genotype groups: TT (major homozygote), CT (heterozygote) and CC (minor homozygote). The y axis is the total IgE level on a logarithmic scale. Plot was created in R using the box plot function from the graphics package.
(0.38 MB TIF)

Figure S2 Patterns of pairwise LD between the SNPs at the RAD50-IL13 locus.
(0.03 MB TIF)

Table S1 Description of study populations.
(0.05 MB DOC)

Table S2 KORA S3/F3 500K SNP exclusion. Detailed breakdown of SNPs that were monomorphic or did not pass quality control and therefore did not enter analysis.
(0.04 MB DOC)

Table S3 Details on the association analysis of SNPs selected for replication (additive model).
(0.25 MB DOC)

Table S4 Genotyping details on replication and fine-mapping stages.
(0.15 MB DOC)

Table S5 Primers used to amplify the exons of *FCER1A*.
(0.04 MB DOC)

Table S6 Mutational analysis of *FCER1A* exons.
(0.04 MB DOC)

Table S7 Associations between *FCERA1* haplotypes and IgE levels in KORA S4. Results correspond to the single SNP analyses where presence of A (rs2427837) and C (rs2251746) alleles at respective positions were strongly associated.
(0.05 MB DOC)

Table S8 Association analysis of FCERA1 and RAD50 variants with AE in 562 German AE trios and with asthma in 638 UK asthma cases and 633 controls.
(0.06 MB DOC)

Table S9 Extended SNP analysis in the RAD50-IL13 region in a subset of 526 children from the ISAAC replication cohort and association with total IgE levels.
(0.05 MB DOC)

Table S10 Genes that have been associated with total IgE ordered by their chromosomal position.
(0.16 MB DOC)

Table S11 Affymetrix SNPs in selected candidate genes for total IgE, which yielded a nominal p-value <0.05 in the GWAS. Genes are ordered by their chromosomal position.
(0.14 MB DOC)

Text S1 Supplementary information.
(0.10 MB DOC)
