# Supplementary figures and images for: Genome-Wide Scan on Total Serum IgE Levels Identifies FCER1A as Novel Susceptibility Locus
Source: PLoS Genet. 2008 Aug 22;4(8):e1000166. doi: 10.1371/journal.pgen.1000166 (PMC2565692; doi:10.1371/journal.pgen.1000166)

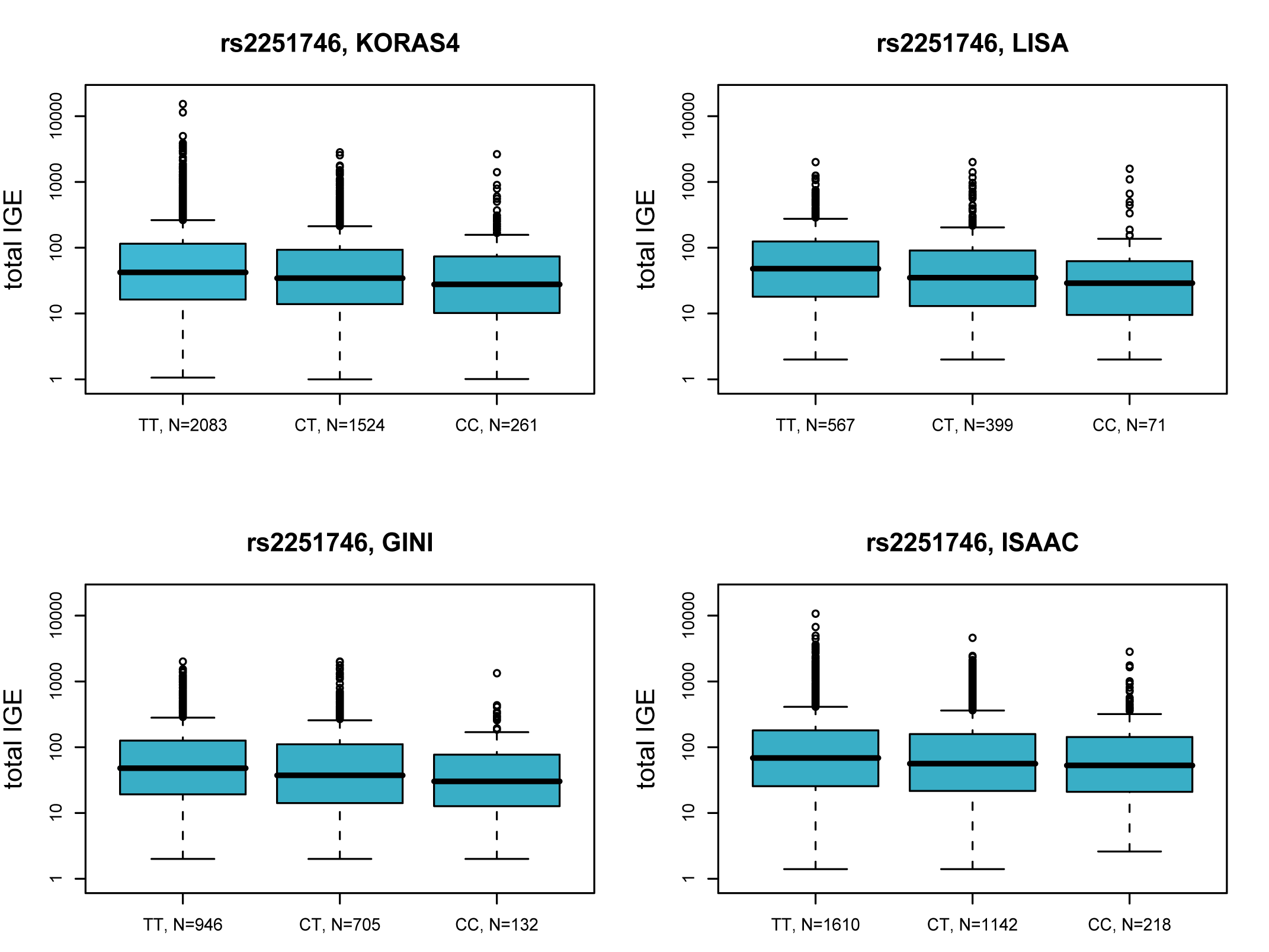

Supplement: Figure S1 — Box plot comparing the total IgE levels for the genotypes at rs2251746. The x axis represents the three genotype groups: TT (major homozygote), CT (heterozygote) and CC (minor homozygote). The y axis is the total IgE level on a logarithmic scale. Plot was created in R using the box plot function from the graphics package. (0.38 MB TIF) [file pgen.1000166.s001.tif]

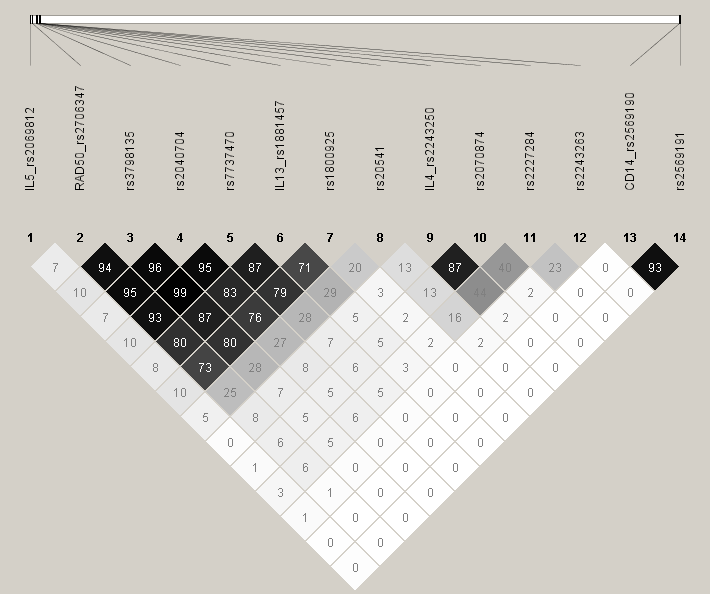

Supplement: Figure S2 — Patterns of pairwise LD between the SNPs at the RAD50-IL13 locus. (0.03 MB TIF) [file pgen.1000166.s002.png]
