# Supplementary material for: Genome-Wide Scan on Total Serum IgE Levels Identifies FCER1A as Novel Susceptibility Locus
Source: PLoS Genet. 2008 Aug 22;4(8):e1000166. doi: 10.1371/journal.pgen.1000166 (PMC2565692; doi:10.1371/journal.pgen.1000166)
Supplement: Table S1 — Description of study populations. (0.05 MB DOC) [file pgen.1000166.s003.doc]

|  | **GWAS** | **Replication samples** | | | |
| --- | --- | --- | --- | --- | --- |
|  | **KORA S3/F3 500K** | **S4** | **LISA** | **GINI** | **ISAAC** |
| **Number total** | 1530 | 3890 | 1042 | 1839 | 2998 |
| **Number males** | 758 (49.5%) | 1927 (49.5%) | 565 (54.2%) | 933 (50.7%) | 1510 (50.4%) |
| **Number females** | 772 (50.5%) | 1963 (50.5%) | 477 (45.8%) | 906 (49.3%) | 1488 (49.6%) |
| **Mean age (range)** | 52.3 (25-69) | 49.1 (25-74) | 6.0 | 6.0 | 9.6 (8-12) |
| **Geometric mean total IgE in kU/L (95% CI)** |  |  |  |  |  |
| **Total** | 42.41  (39.56 - 45.47) | 38.88  (37.17 - 40.68) | 39.97  (36.63 -- 43.62) | 43.92  (41.18-46.85) | 68.92  (65.50-72.52) |
| **Men** | 54.10  (49.01 - 59.72) | 49.01  46.04 - 52.18) | 49.53  (43.82 - 55.98) | 50.17  (45.77 - 54.99) | 77.56  (72.08-83.46) |
| **Women** | 33.40  (30.36 - 36.74) | 30.98  (29.08 - 33.00) | 31.01  (27.49-34.98) | 38.30  (35.01 - 41.90) | 61.14  (56.99- 65.58) |
