# Supplementary material for: Genome-Wide Scan on Total Serum IgE Levels Identifies FCER1A as Novel Susceptibility Locus
Source: PLoS Genet. 2008 Aug 22;4(8):e1000166. doi: 10.1371/journal.pgen.1000166 (PMC2565692; doi:10.1371/journal.pgen.1000166)
Supplement: Table S2 — KORA S3/F3 500K SNP exclusion. Detailed breakdown of SNPs that were monomorphic or did not pass quality control and therefore did not enter analysis. (0.04 MB DOC) [file pgen.1000166.s004.doc]

| **Reason for exclusion** | **Number of SNPs excluded** |
| --- | --- |
| Monomorphic | 17415 |
| HWE (deviation from HWE (P-value < 10^-6^)) | 4527 |
| MAF (minor allele frequency < 0.03) | 64850 |
| Genotyping efficiency (GE < 0.95) | 35904 |
| HWE + MAF | 346 |
| HWE + GE | 8405 |
| MAF + GE | 4972 |
| HWE + MAF + GE | 44 |
| **Total** | 136463 |
