# Supplementary material for: Genome-Wide Scan on Total Serum IgE Levels Identifies FCER1A as Novel Susceptibility Locus
Source: PLoS Genet. 2008 Aug 22;4(8):e1000166. doi: 10.1371/journal.pgen.1000166 (PMC2565692; doi:10.1371/journal.pgen.1000166)
Supplement: Table S3 — Details on the association analysis of SNPs selected for replication (additive model). (0.25 MB DOC) [file pgen.1000166.s005.doc]

|  |  |  |  | **GWAS (KORA S3/F3 500K)**  **(n=1530)** | | | | **Replication KORA S4**  **(n=3890)** | | | |
| --- | --- | --- | --- | --- | --- | --- | --- | --- | --- | --- | --- |
| **rs number** | **Position*** | **Gene Structure*** | **Gene*** | **MAF** | **Estimate** | **P value** | **Genotyping efficiency** | **MAF** | **Estimate** | **P value** | **Genotyping efficiency** |
| rs2427837 | chr1:157525169 | 5’ region | FCER1A | 26.57% | -0.235 | 6.19E-05 | 95.68% | 27.42% | -0.212 | 3.23E-09 | 99.24% |
| rs12368672 | chr12:55798737 | 5’ region | STAT6 | 38.25% | 0.167 | 8.52E-04 | 99.39% | 38.00% | 0.156 | 2.03E-06 | 99.53% |
| rs2706347 | chr5:131933016 | intronic | RAD50 | 23.70% | 0.236 | 4.05E-05 | 99.09% | 22.14% | 0.143 | 0.000225629 | 98.60% |
| rs7737470 | chr5:132001962 | intronic | RAD50 | 24.22% | 0.231 | 4.81E-05 | 99.94% | 22.56% | 0.142 | 0.000226596 | 99.26% |
| rs3798135 | chr5:131993008 | intronic | RAD50 | 24.38% | 0.227 | 6.58E-05 | 99.82% | 22.67% | 0.142 | 0.000231899 | 99.61% |
| rs2040704 | chr5:132001076 | intronic | RAD50 | 24.59% | 0.221 | 9.25E-05 | 99.70% | 23.06% | 0.140 | 0.000246599 | 99.11% |
| rs17500878 | chr4:38379324 |  |  | 28.68% | 0.195 | 0.000259962 | 99.70% | 28.09% | 0.102 | 0.004851719 | 98.06% |
| rs4345891 | chr10:1704379 | intronic | ADARB2 | 25.51% | -0.213 | 0.000174564 | 99.21% | 27.65% | -0.094 | 0.009460605 | 99.61% |
| rs7735200 | chr5:178335013 |  |  | 16.07% | -0.279 | 3.09E-05 | 99.94% | 16.09% | 0.096 | 0.026525431 | 99.80% |
| rs17411897 | chr15:48070212 | intronic | ATP8B4 | 3.90% | 0.500 | 8.48E-05 | 99.76% | 3.62% | -0.187 | 0.031437837 | 99.56% |
| rs10863711 | chr1:204812348 | intronic | RASSF5 | 43.24% | 0.182 | 0.00024168 | 99.88% | 42.06% | -0.064 | 0.048615797 | 98.92% |
| rs10738507 | chr9:18304001 |  |  | 47.20% | 0.178 | 0.000306891 | 96.72% | 46.73% | -0.062 | 0.054093381 | 99.53% |
| rs2833584 | chr21:32258059 | intronic | HUNK | 10.77% | 0.351 | 7.96E-06 | 96.29% | 10.41% | 0.101 | 0.057010423 | 99.43% |
| rs2801020 | chr10:42277336 |  |  | 18.73% | -0.243 | 0.000126407 | 99.70% | 19.30% | -0.058 | 0.150731057 | 99.83% |
| rs4867943 | chr5:169558250 |  |  | 31.17% | 0.193 | 0.000432635 | 98.36% | 31.51% | 0.052 | 0.154633138 | 90.78% |
| rs2520094 | chr9:135909806 | intronic | BRD3 | 30.79% | 0.198 | 0.000351965 | 95.13% | 30.27% | 0.047 | 0.175791773 | 99.80% |
| rs10771978 | chr12:32717095 |  |  | 27.10% | -0.214 | 0.000165691 | 100.00% | 26.88% | 0.047 | 0.18740227 | 96.95% |
| rs2248775 | chr1:69469896 |  |  | 27.28% | 0.185 | 0.00068095 | 100.00% | 27.08% | 0.046 | 0.204922228 | 99.51% |
| rs4943387 | chr13:36004633 |  |  | 28.03% | -0.198 | 0.000325101 | 99.51% | 27.54% | 0.044 | 0.232691386 | 99.58% |
| rs829612 | chr2:30484591 |  |  | 43.93% | 0.179 | 0.000275827 | 99.64% | 44.13% | -0.038 | 0.239047104 | 99.53% |
| rs2508756 | chr11:75921549 | intronic | C11orf30 | 44.34% | -0.189 | 0.000137891 | 99.88% | 43.44% | -0.037 | 0.257370461 | 99.21% |
| rs3768513 | chr1:157278841 | intronic | IFI16 | 14.46% | 0.282 | 6.53E-05 | 99.51% | 13.75% | -0.051 | 0.282437895 | 99.26% |
| rs2273758 | chr1:114082240 | intronic | PHTF1 | 39.62% | 0.168 | 0.000681559 | 98.48% | 38.94% | -0.035 | 0.293351985 | 99.53% |
| rs1956849 | chr14:98034246 |  |  | 33.08% | -0.200 | 0.000117626 | 98.30% | 34.67% | 0.034 | 0.304748531 | 98.18% |
| rs2277615 | chr17:40290220 | intronic | EFTUD2 | 32.36% | 0.220 | 4.67E-05 | 99.33% | 33.51% | -0.035 | 0.308459254 | 99.68% |
| rs17628255 | chr17:40304977 | intronic | EFTUD2 | 12.05% | -0.268 | 0.000375127 | 98.18% | 12.65% | 0.027 | 0.565723563 | 90.90% |
| rs849538 | chr2:206296221 | intronic | NRP2 | 48.84% | 0.206 | 3.03E-05 | 99.70% | 48.38% | 0.033 | 0.309237792 | 99.53% |
| rs849540 | chr2:206294712 | intronic | NRP2 | 49.60% | 0.208 | 2.19E-05 | 100.00% | 49.44% | 0.031 | 0.328662965 | 99.46% |
| rs4380643 | chr5:132273842 | intronic | AFF4 | 30.21% | 0.261 | 1.57E-06 | 99.27% | 29.96% | 0.035 | 0.312424707 | 99.88% |
| rs10479013 | chr5:132272685 | intronic | AFF4 | 20.42% | 0.253 | 6.95E-05 | 95.01% | 19.98% | 0.032 | 0.421161712 | 99.46% |
| rs6702619 | chr1:99818834 |  |  | 49.03% | 0.176 | 0.00034666 | 100.00% | 49.46% | -0.032 | 0.31344827 | 99.43% |
| rs8019638 | chr14:50900116 |  |  | 9.45% | 0.361 | 3.67E-05 | 95.92% | 10.60% | -0.047 | 0.336200308 | 98.94% |
| rs1326099 | chr13:58786055 |  |  | 36.53% | -0.201 | 9.73E-05 | 97.51% | 37.36% | -0.032 | 0.337439528 | 99.73% |
| rs1410269 | chr13:58733622 |  |  | 37.41% | -0.194 | 0.000226089 | 96.41% | 38.73% | -0.031 | 0.351171139 | 99.70% |
| rs2037010 | chr3:165730724 |  |  | 37.58% | -0.187 | 0.00019371 | 99.88% | 38.66% | 0.031 | 0.353456645 | 98.11% |
| rs787633 | chr10:95109238 | intronic | FER1L3 | 28.86% | 0.186 | 0.000645271 | 99.39% | 27.03% | 0.033 | 0.372385463 | 99.53% |
| rs17123958 | chr14:51011874 |  |  | 3.18% | 0.558 | 6.77E-05 | 99.57% | 3.89% | -0.073 | 0.382256705 | 99.14% |
| SNP_A-1782550^#^ | chr4:83698942 | intronic | FLJ12993 | 10.01% | 0.297 | 0.000359165 | 98.78% | 10.67% | 0.044 | 0.401044371 | 99.83% |
| rs2922309 | chr2:177188300 |  |  | 15.10% | -0.258 | 0.000221428 | 99.70% | 12.81% | 0.040 | 0.407423846 | 99.61% |
| rs481689 | chr5:84812541 |  |  | 20.85% | 0.251 | 4.70E-05 | 99.76% | 19.58% | 0.034 | 0.408848387 | 99.66% |
| rs7328732 | chr13:27714278 | intronic | PAN3 | 29.75% | 0.186 | 0.00051414 | 99.88% | 29.47% | -0.027 | 0.438666196 | 99.24% |
| rs2001011 | chr7:141616055 |  |  | 26.65% | -0.192 | 0.000533399 | 99.27% | 25.94% | -0.028 | 0.446796897 | 98.33% |
| rs6464482 | chr7:141609452 |  |  | 26.52% | -0.188 | 0.000702127 | 99.76% | 25.90% | -0.028 | 0.448583256 | 94.86% |
| rs990157 | chr13:53279249 |  |  | 34.89% | -0.181 | 0.000456037 | 99.64% | 33.94% | -0.027 | 0.456490645 | 90.88% |
| rs6452647 | chr5:84576005 |  |  | 21.13% | 0.240 | 5.18E-05 | 99.88% | 28.84% | 0.021 | 0.461247955 | 99.66% |
| rs10793491 | chr10:43598732 |  |  | 23.77% | 0.239 | 2.79E-05 | 98.54% | 25.27% | 0.027 | 0.465259604 | 98.99% |
| rs4392995 | chr9:27826571 |  |  | 3.13% | 0.571 | 5.94E-05 | 100.00% | 2.57% | -0.064 | 0.539416773 | 97.07% |
| rs6651173 | chr8:133236822 | intronic | KCNQ3 | 45.65% | 0.196 | 6.42E-05 | 100.00% | 47.05% | 0.020 | 0.542785448 | 99.48% |
| rs7526120 | chr1:106451947 |  |  | 6.92% | -0.364 | 0.00014804 | 99.82% | 6.86% | -0.040 | 0.549785326 | 90.88% |
| rs16868519 | chr5:16425052 |  |  | 10.65% | -0.327 | 2.88E-05 | 99.64% | 10.55% | 0.030 | 0.555169543 | 99.43% |
| rs3907223 | chr5:16434517 |  |  | 10.69% | -0.319 | 4.39E-05 | 99.88% | 10.81% | 0.030 | 0.557570801 | 99.80% |
| rs9474972 | chr6:54694596 |  |  | 41.36% | 0.179 | 0.00038587 | 99.94% | 42.33% | 0.019 | 0.5638028 | 99.51% |
| rs10114741 | chr9:27792613 |  |  | 3.13% | 0.560 | 8.67E-05 | 99.21% | 2.68% | -0.055 | 0.581560391 | 98.82% |
| rs951260 | chr10:43520976 |  |  | 23.54% | 0.237 | 3.25E-05 | 99.88% | 25.18% | 0.020 | 0.584192725 | 99.58% |
| rs12516809 | chr5:16461984 |  |  | 10.51% | -0.339 | 1.90E-05 | 98.36% | 10.68% | 0.028 | 0.587300136 | 99.34% |
| rs7980576 | chr12:32983423 |  |  | 43.52% | 0.172 | 0.000479738 | 99.94% | 43.79% | -0.017 | 0.587542252 | 99.04% |
| rs17519439 | chr13:26541135 | exonic | USP12 | 8.16% | -0.394 | 1.75E-05 | 97.26% | 7.88% | -0.031 | 0.60362714 | 99.11% |
| rs460175 | chr9:97517939 |  |  | 25.23% | -0.216 | 0.000102464 | 99.57% | 24.07% | -0.019 | 0.60602295 | 98.33% |
| rs9287421 | chr2:133030315 | intronic | GPR39 | 16.88% | 0.252 | 0.000151598 | 99.64% | 17.17% | -0.022 | 0.611104018 | 99.04% |
| rs10492336 | chr12:113069963 |  |  | 34.40% | -0.183 | 0.000315143 | 98.84% | 34.38% | -0.016 | 0.643737179 | 99.48% |
| rs7861951 | chr9:27808130 |  |  | 3.11% | 0.563 | 8.32E-05 | 99.70% | 2.74% | -0.044 | 0.655983548 | 99.61% |
| rs1230658 | chr1:114019974 | intronic | MAGI3 | 23.72% | 0.222 | 9.70E-05 | 100.00% | 24.84% | -0.016 | 0.663736739 | 99.46% |
| rs1217200 | chr1:113962639 | intronic | MAGI3 | 24.27% | 0.234 | 4.72E-05 | 95.50% | 24.58% | -0.015 | 0.68833338 | 94.56% |
| rs12515725 | chr5:168523878 | intronic | SLIT3 | 43.48% | 0.207 | 3.61E-05 | 99.76% | 43.62% | 0.013 | 0.690861139 | 99.70% |
| rs157936 | chr7:130236163 |  |  | 28.66% | 0.229 | 2.91E-05 | 96.90% | 27.56% | 0.014 | 0.700046468 | 94.86% |
| rs7574303 | chr2:128357845 | intronic | AMMECR1L | 12.16% | -0.303 | 4.60E-05 | 98.54% | 11.53% | 0.019 | 0.704428723 | 99.26% |
| rs11139093 | chr9:83057977 |  |  | 29.47% | 0.200 | 0.000521885 | 97.63% | 30.61% | -0.013 | 0.716678564 | 99.70% |
| rs2931116 | chr5:84639708 |  |  | 20.87% | 0.236 | 9.75E-05 | 99.51% | 20.51% | 0.014 | 0.734486799 | 99.53% |
| rs1602452 | chr4:130781241 |  |  | 8.80% | -0.308 | 0.00042454 | 99.51% | 8.58% | 0.019 | 0.751626083 | 90.97% |
| rs7215172 | chr17:40030029 |  |  | 50.00% | 0.184 | 0.000181668 | 99.88% | 49.56% | 0.011 | 0.753000567 | 91.20% |
| rs10090180 | chr8:29475483 |  |  | 12.45% | 0.294 | 0.000129093 | 95.50% | 12.15% | 0.015 | 0.759514839 | 99.68% |
| rs12341214 | chr9:27811861 |  |  | 3.16% | 0.569 | 6.45E-05 | 99.15% | 2.69% | -0.030 | 0.762393969 | 99.34% |
| rs1591000 | chr6:78808445 |  |  | 34.52% | -0.209 | 6.26E-05 | 99.64% | 36.25% | -0.010 | 0.765918278 | 99.53% |
| rs731955 | chr13:98668641 | intronic | PHGDHL1 | 37.75% | -0.193 | 0.000286722 | 95.07% | 37.98% | -0.010 | 0.769839524 | 99.78% |
| rs4840947 | chr8:8269553 |  |  | 29.62% | -0.199 | 0.000240051 | 98.78% | 32.05% | 0.007 | 0.826590489 | 99.73% |
| rs1953289 | chr13:81015510 |  |  | 9.14% | 0.288 | 0.000590014 | 99.51% | 7.99% | 0.013 | 0.829028779 | 97.84% |
| rs4293602 | chr2:48586448 | intronic | CCDC128 | 27.18% | 0.209 | 0.000188381 | 99.94% | 28.16% | -0.007 | 0.844508489 | 99.29% |
| rs10510391 | chr3:8500794 |  |  | 10.93% | -0.267 | 0.00057424 | 99.94% | 10.10% | -0.008 | 0.876882235 | 99.68% |
| rs6897714 | chr5:84626651 |  |  | 21.21% | 0.237 | 9.70E-05 | 98.36% | 20.44% | 0.004 | 0.927041041 | 98.84% |
| rs6748436 | chr2:30453138 |  |  | 22.44% | 0.213 | 0.000328849 | 98.11% | 23.34% | -0.002 | 0.950180315 | 98.77% |
| rs7229385 | chr18:67508569 |  |  | 44.74% | 0.198 | 5.35E-05 | 99.94% | 43.59% | -0.002 | 0.96217847 | 99.73% |
| rs2821195 | chr9:11679891 |  |  | 35.60% | 0.199 | 0.000114315 | 99.70% | 36.93% | -0.002 | 0.963397047 | 99.70% |
| rs7541801 | chr1:101813107 |  |  | 43.95% | -0.188 | 0.000183747 | 100.00% | 43.66% | -0.001 | 0.98458379 | 98.48% |
| rs4450260 | chr13:108001372 |  |  | 38.70% | -0.198 | 0.000117232 | 99.57% | 40.55% | 0.000 | 0.993558965 | 96.83% |

# Affymetrix 500k probe set ID, rs number not available

* HapMap data release #22, April 2007, on NCBI B36 assembly, dbSNP b126
