# Supplementary material for: Genome-Wide Scan on Total Serum IgE Levels Identifies FCER1A as Novel Susceptibility Locus
Source: PLoS Genet. 2008 Aug 22;4(8):e1000166. doi: 10.1371/journal.pgen.1000166 (PMC2565692; doi:10.1371/journal.pgen.1000166)
Supplement: Table S4 — Genotyping details on replication and fine-mapping stages. (0.15 MB DOC) [file pgen.1000166.s006.doc]

| **rs number** | **2nd primer 5’-3’** | **1st primer 5’-3’** | **Extension primer 5’-3’** |
| --- | --- | --- | --- |
| rs10090180 | ACGTTGGATGTATGTCAGTCTCCTTGGTCC | ACGTTGGATGTTGCTTGGCCTGAGAAGATG | tCACAGAGCTCCTTTGG |
| rs10114741 | ACGTTGGATGCTGAGATATCCTGTTACGCC | ACGTTGGATGACTGAGGCGTTTTCTTACCC | ctagATCCTGTTACGCCCCTGTAGAG |
| rs10479013 | ACGTTGGATGGGAGTGTGAAACTCCATCTC | ACGTTGGATGCAGCCGCTCAAGCATATCTT | TGTTTTAACAATGAATGAAGTAAC |
| rs10489854 | ACGTTGGATGCAAGGAGGTCAAGAATGCCC | ACGTTGGATGATACCCAGACTATGACTGAC | ggCAAGAATGCCCTTCACC |
| rs10492336 | ACGTTGGATGTCAGTTCTAACCATTATGGC | ACGTTGGATGCAGCCTAACCTTTCAGAATT | GCAATGTTTATTATGAGATAGCCC |
| rs10510391 | ACGTTGGATGCAGGCATGGGCAATATTATG | ACGTTGGATGTTAGCTGACCACCTGTTCTG | gATCAACTTCTAAAAAAGGCATTGTA |
| rs10738507 | ACGTTGGATGTACGTTGTCCTTGGAGAAAC | ACGTTGGATGCAACTTAGTAGCAAAACACC | tTTCAAGTCCTTTGCCCATTTTTTTTA |
| rs10771978 | ACGTTGGATGCTGCCTCGTGTATTTATTCC | ACGTTGGATGCCAGCAAAATATACTGCCTG | gTGATGAAGTACTTGTTAGGCATA |
| rs10793491 | ACGTTGGATGTCTTGGGCTAGGAGACAATG | ACGTTGGATGAGTCTTAGGTCTGAGCTGTG | gatCCTTTCTATGAAACCTCCA |
| rs10863711 | ACGTTGGATGTCTTCTGTAAAGCTGTGCCC | ACGTTGGATGAGTTGTGTCCTAGGCCACAG | CTGTGCCCATGTACTC |
| rs11139093 | ACGTTGGATGATGACAAGGTAAGTACAGAC | ACGTTGGATGCACATGTTTGGATGTTGCAG | GAGAGTGTTCATAAACTTTCA |
| rs1217200 | ACGTTGGATGTAATTGTTGGTGTTCACTC | ACGTTGGATGTTGGGCGACAAGAGTGAAAC | tTTGGTGTTCACTCATATCA |
| rs1230658 | ACGTTGGATGCCTGTCTGATTTCCAAACCC | ACGTTGGATGTACAGCTCGTCAAGAACTGC | ACCCATGTTCTTGTCAC |
| rs12341214 | ACGTTGGATGCTTGAAGACAGGGTGGGAGA | ACGTTGGATGTGCCCAGCACTGAATGAATG | aAGGGTGGGAGATGAAGA |
| rs12368672 | ACGTTGGATGATCTCCTTGCTTGAAGCCTG | ACGTTGGATGAAGGAGGAATGTTCTCAAGG | GGCTTGTCTGGAACCT |
| rs12515725 | ACGTTGGATGAGCCAATGGGAGGGAAAATG | ACGTTGGATGTAGAATGGGACCTTGGGAAG | CCGGCTAATTCAATTCC |
| rs12516809 | ACGTTGGATGACAGTCTCCTTTCTCTGAGC | ACGTTGGATGGTAAAGTAGTTAACTTTCTTC | GGTTCTTCTGAAAGAAATGTATTAAA |
| rs12565775 | ACGTTGGATGACTTGGTTGATTTCTCCCCC | ACGTTGGATGGCTGTAATAACTGGGTAGAC | cccaCAATTCTAGTACTTCCCTTAAAT |
| rs1326099 | ACGTTGGATGAGCTAATGGCCTCCATTTCC | ACGTTGGATGGATGCAGGCAGTGGAAAGTT | AGAAGGTGCCACATGG |
| rs1410269 | ACGTTGGATGTGCCATATTGTCTATGCAGC | ACGTTGGATGCCTTAGCTGGATCCTATCTG | TCTCATTTTTTAGCATCATCAT |
| rs157936 | ACGTTGGATGCTCAAGAGGTCAAAGTAGGC | ACGTTGGATGCTGGCACACAGTAGGTGTTC | ttggTCATCCATTTATTCAGCAAA |
| rs1591000 | ACGTTGGATGGGAAAACCACATTATTCCCC | ACGTTGGATGGGGATGTAGAGTGTAAAAG | TGGCCAGTCTGCAAAT |
| rs1602452 | ACGTTGGATGAGATGTCCATTTCCTGCCTC | ACGTTGGATGGGAAGAAAGATGAAGAAAGC | GCCTCAGATTTGGGGAC |
| rs16868519 | ACGTTGGATGTTGCCCATGATTCTATCATC | ACGTTGGATGGGTTTCAGTCTGAAATGTTG | TTCTATCATCTTGGTAATATAACTG |
| rs17123958 | ACGTTGGATGGCTAAGAACACTCTCTGAAG | ACGTTGGATGGGAAGCACTATGATGTCAGG | TGGGTGAAATCAGCTC |
| rs17411897 | ACGTTGGATGAAAAATGGAACTCAGTGCCC | ACGTTGGATGTAGGAAGAGATGAAGCCAGC | ACTCAGTGCCCAATAGTCTA |
| rs17500878 | ACGTTGGATGCTGCAGACAATCCAGCAAAA | ACGTTGGATGTCTTTTTGGAAGCAGGCAGG | acacTCCAGCAAAAATCTACAAACTC |
| rs17519439 | ACGTTGGATGACCCCCAAGGAATTCAAAGC | ACGTTGGATGGCTCCTCTTTTAAAACAGCC | GGTGCAATTAATAAAGAGAAAATG |
| rs17628255 | ACGTTGGATGTGCAAGGTCACCATGAGTTC | ACGTTGGATGTCCCTCACTGTTAGTGCTTC | ggTTGATGTCTGCCTCCATAC |
| rs17772565 | ACGTTGGATGCAGTCTAGTGCCTTAGCATC | ACGTTGGATGTTATTCCTGCCTTGACTGCC | gggGTGCCTTAGCATCAACAAG |
| rs17772583 | ACGTTGGATGTTGTGTATTACAGAACCCTC | ACGTTGGATGCCAGAGTGCTTTACTTAGCG | gattcCAGAACCCTCATAGCATG |
| rs1953289 | ACGTTGGATGATAGCTCAATACCCAAATGC | ACGTTGGATGTTATGCATTGGGGATTAGAC | cAATGCTATAAATTCACACATTTTCTT |
| rs1956849 | ACGTTGGATGCTGTGCCTCTGGAAAACAGC | ACGTTGGATGGCAAGACATGCTGAAAGCAC | TGGAAAACAGCAAGTCC |
| rs2001011 | ACGTTGGATGGGGTTGAGTGAAGCAGGTGT | ACGTTGGATGACCTACCCTGACTTTGATGC | tgatTGAAGCAGGTGTTGATAG |
| rs2037010 | ACGTTGGATGACACATGCACACATACGCAC | ACGTTGGATGGAGTTTTGGCTGCTTCTAGG | ccccCACACACATTTCATAACTTTT |
| rs2040704 | ACGTTGGATGAAAGAGTTTCCTCTGGATGG | ACGTTGGATGCTGTCATAGTGTAGATAGGG | TTCCTCTGGATGGTTTTTCTTAT |
| rs2069812 | ACGTTGGATGCAAGATGATGTCCAGACTCC | ACGTTGGATGCAATCAGAAAAGCACAGCTTG | GTCCAGACTCCTGGATCT |
| rs2214370 | ACGTTGGATGGAAGTTCTCACTAAAGCAGG | ACGTTGGATGATCCCAGGCGAAGGAGTAAG | tGTATAAATATTTTATTGAGTTTTCCTT |
| rs2240032 | ACGTTGGATGCTGCTGAGCTCTCCTTCTG | ACGTTGGATGGGTCAAAGGAGGTGTTTCAG | acccgCTGCCAGAAAGGGACT |
| rs2248775 | ACGTTGGATGTTACTTCCAGAGGACAAGGC | ACGTTGGATGTTCACAGCCGTGTTGGTTTC | gcTTTGCCTGATAAGACAATGAT |
| rs2251746 | ACGTTGGATGAGGCACAGCTGATGGGTTAA | ACGTTGGATGCTGGAGAGATCTAAGGCTTC | GATGGGTTAACCAGATATGA |
| rs2273758 | ACGTTGGATGAGCAGCAAAAAGAAACTGTC | ACGTTGGATGCTTTGTTTGGCTGTTACTAC | tAAACTGTCATTGCTTATTCC |
| rs2277615 | ACGTTGGATGTTACTCTTGGCTCAGGAATC | ACGTTGGATGCAACAGCAGGTTAAAAGAGC | GGGCTTTTTGGTGATG |
| rs2427824 | ACGTTGGATGTGTTGTTCAGAGACAGGTGG | ACGTTGGATGAGGGTTGTTCTGTGTTTGGC | gggcGAGACAGGTGGACAAAA |
| rs2427827 | ACGTTGGATGCACCTGGCATATGTTTGGTA | ACGTTGGATGCAACTTAGAAAAGTGGGATGC | cccgcTGCTGCTGTTTTATTCTGC |
| rs2427837 | ACGTTGGATGCTCTCTGTGTTACTACCTGG | ACGTTGGATGCAGGTGCCCTCAAGGATTAT | aaGACTTTCTCCCCATGATG |
| rs2494262 | ACGTTGGATGAGCCTTCAGGTTCTACCTTC | ACGTTGGATGCCAAGTCTCCTGAGATCATC | ctacaTTCCTTACCTCTTAGTAGCATG |
| rs2508756 | ACGTTGGATGTGGATTTATTCCCCACCCTC | ACGTTGGATGGACATTGCTTTGAGTAAGTC | CCCACCCTCACCTTGA |
| rs2511211 | ACGTTGGATGCAGTGTCAAAGTTGTATACAG | ACGTTGGATGCTCAACTGATCTAACCCAGG | gggagACAGAATAGCTTTCTGTAAGTAC |
| rs2520094 | ACGTTGGATGTTGAAGCCCTTTCCTCTCTG | ACGTTGGATGCAAGCTGGATGCTCAATGTC | caaGTGGGAGGAAGTGAGG |
| rs2706347 | ACGTTGGATGCAGAAGACATCAGAACTAGG | ACGTTGGATGAGAATTTTTGTGTCCTTGC | AGGGAAAATGAAACATTTTATGATGA |
| rs2801020 | ACGTTGGATGTATCTGCTTTCAGCTCCAGG | ACGTTGGATGCCCCATTTTAAGACATCAGG | CTCCAGGTTGTAGCTATT |
| rs2821195 | ACGTTGGATGCCACACACATCCTGTTTGTC | ACGTTGGATGATATGTAGGCTTGGAGATAG | TGTCACTTCTTATTCACCA |
| rs2833584 | ACGTTGGATGTTCTGTGGATTCACAAGGGC | ACGTTGGATGTCTATTAATTCTTCGGCTC | ATGCTGTTTTCAACTCAAG |
| rs2922309 | ACGTTGGATGAGTAATTCACTGGATGGCCC | ACGTTGGATGTTCTCAGTTTAGCACAGGAC | tCCCTCTCTCTCACAAAC |
| rs2931116 | ACGTTGGATGTGTTTGTCGTGTAGGGATGG | ACGTTGGATGGTGTTATAAAGTTGTCTGGTC | GGATGGAGAAAACAGGCATATT |
| rs3768513 | ACGTTGGATGCTGATGTATGGTGAGAGAGC | ACGTTGGATGTCCCCCAGTATTACTATACC | AAGGAGAAAGGATCGCAAAG |
| rs3798135 | ACGTTGGATGAAAGCACTCCTGTACCTCTC | ACGTTGGATGGAAAAGTGCCTCAGTCACAG | CTCCACCAAGCTCCCTCATAG |
| rs3845625 | ACGTTGGATGGAAAAGGAAGAGGATAGTGG | ACGTTGGATGTATACCAGCTGACAAATGAC | ATAGTGGAATATCTGTTTACTTT |
| rs3907223 | ACGTTGGATGGCATGGATAAGGGATTCTTTC | ACGTTGGATGCTGAAACCAGGTCAGTGAAG | TATCATGGATTTTAATACAGCAA |
| rs4293602 | ACGTTGGATGATCTCCATGGCAAGTCACTC | ACGTTGGATGCTCTGGTTCTGTTTTGATGG | GCAATTACAAAAACAGGC |
| rs4345891 | ACGTTGGATGTGGACAGCCTGCCATCGTTT | ACGTTGGATGGCTGCAAAGGAGGTTGAAAG | GCCATCGTTTTCCTCT |
| rs4380643 | ACGTTGGATGCACATGGAACAGAGGGATTG | ACGTTGGATGAGGATCATATTGGGGAGCTG | AATTGTTTTTGTTTTTAAATGGGA |
| rs4392995 | ACGTTGGATGGCAGTGTTTCTCCCTTGGTC | ACGTTGGATGCCATCATCTGCACAGAGATA | ccctTGTTTCTCCCTTGGTCTGTGATA |
| rs4450260 | ACGTTGGATGTGAAGGAAAGCAAGGAGGAC | ACGTTGGATGCCCCTTGTGCCTTTTTATAG | ccACAATGTTGTTTCGGTATC |
| rs460175 | ACGTTGGATGCTGTCTGGAAATCAAGTTGC | ACGTTGGATGAACACAGTAAACTCAGATG | tcacGTTGCATTTTTCCTTCCCCATG |
| rs481689 | ACGTTGGATGGGGTGCTTGTTTGTAGTATC | ACGTTGGATGGGTTAAACCATTGGTTGTTG | AGTATCTGCTGATATCTCTGG |
| rs4840947 | ACGTTGGATGTTTAAATCCATCCCTGGCCC | ACGTTGGATGAGTCCCCGAATGAATGAGAG | CCTGGCCCATAGTGAG |
| rs4867943 | ACGTTGGATGTTCTCAACAGGCAAGTCAGG | ACGTTGGATGGCCGATCAATGGCAAATACG | agGTCAGGAGCTGCTAAGATAAA |
| rs4943387 | ACGTTGGATGACAGCATAGAACAGTGCTGG | ACGTTGGATGCATTTGTCCATCTATCCCTG | GTGCTGGCATAGGGTT |
| rs6452647 | ACGTTGGATGGCAACTACTTGGCTACTGTG | ACGTTGGATGCCATTCCTGTTAAGAGGCAC | atCTATCTCTTACTCCAGGCAAT |
| rs6464482 | ACGTTGGATGTCACAGGTAGAGATAGAGGC | ACGTTGGATGGCTTTCCCCCACCTTTTTTC | atAGGCTGTAAATAATAAGGTCTTAC |
| rs6651173 | ACGTTGGATGGTGTTGCAAAGCCAGGATTC | ACGTTGGATGTTTCCAGGTTTTCTTGGCCC | ACTCCTAAACTAGCTCTAC |
| rs6702619 | ACGTTGGATGGAAAGCACTTCCTCCAACTG | ACGTTGGATGTGCTTAAGCAGGACTGAGAG | CTCCTGTGGATCAGTTT |
| rs6748436 | ACGTTGGATGGGAAAACATTTGCATTGCAG | ACGTTGGATGTAGTCAAGCAGATGAGACAG | GCAGTTCAGTTCTATATTCTAATTTAT |
| rs6884762 | ACGTTGGATGGCAGAAAGTTGAGAAGCAAG | ACGTTGGATGTCTCCACTGTAAAGTTACTG | TTTACATAGTCTCAACATTTCT |
| rs6897714 | ACGTTGGATGTGAGCCCAAGCTTGATACTG | ACGTTGGATGGGTTGCAGCAAAGTATTATTC | aTGAAGCACTGGGATGG |
| rs7215172 | ACGTTGGATGTCAGATCCTTCATGCAGGTG | ACGTTGGATGGCAGCAAATTTCCGTGACAG | CAGGTGACGCCACATT |
| rs7229385 | ACGTTGGATGCTATACACCAGGCTTCATGC | ACGTTGGATGCTAGTTGCTGCATCCTCTGG | aATTCAGATTACTTGGGCTTCCA |
| rs731955 | ACGTTGGATGACTTTTCTCTGGCATGCAGG | ACGTTGGATGGGAGGAAAGAGTGAGCTGTA | TGTTTCAGGACTAGGATTT |
| rs7328732 | ACGTTGGATGGCAAACATGAAACAACCCAC | ACGTTGGATGAGAATGTCCTTCATCCCAGC | cccccAACAACCCACTAGACACTA |
| rs7526120 | ACGTTGGATGGGCAGAATCACTCTTGAGAC | ACGTTGGATGGTCAGGCACCTAAAGTTCTC | CCTTAGATATGTAGAGCCA |
| rs7541801 | ACGTTGGATGTAGGAAAGCATGCTGGGTAG | ACGTTGGATGGATAGAGATGAACAGAGGAC | ccTCTGCTCATAGTTTTCTGAGA |
| rs7574303 | ACGTTGGATGTGCAGTTCCTGATCAAAGTC | ACGTTGGATGGGACAAAATGTTCCAAAAGTC | TATTGGTTCTCTTTTCTATAGTTTCA |
| rs7735200 | ACGTTGGATGAATCATGCACCAAGCTGCAC | ACGTTGGATGTGACCACTACAACTGCTCTG | cCTGCACTCTTGAAACATCTTCT |
| rs7737470 | ACGTTGGATGAATGGTGGGAAGCTCTTTGC | ACGTTGGATGGTTGGTTAGCTTCAGGAGAG | cTCAGGCTCAGCTACTA |
| rs7861951 | ACGTTGGATGAGAGTGGATTTAGAGTAGGG | ACGTTGGATGAAGTCACAATGCGTCTCCAG | aAGGGTTTAAGAAATAATCACCTCA |
| rs787633 | ACGTTGGATGGGAAATGATTTTGGCAGCAT | ACGTTGGATGGGGAAGTCAGGGTTTGAATC | gGATTTTGGCAGCATAATCTAGTAGT |
| rs7980576 | ACGTTGGATGGGGTTTCCAGTTATAGGCAG | ACGTTGGATGTGAGCAGTAAATCCAAAGGG | ATAGGCAGGGATAGAGA |
| rs8019638 | ACGTTGGATGATTGTTGGGTTTGGTTTGGG | ACGTTGGATGACCCACAATCTTCCCATCTG | cTTTGCTTTTTCATTTTTTCCTG |
| rs829612 | ACGTTGGATGACGCCTGCAAGATTTCCAC | ACGTTGGATGTGCCGTGGAAGGAGATGAG | CAAGATTTCCACTCTTCAC |
| rs849538 | ACGTTGGATGGTGAAAGATTTGTTCCTCTCC | ACGTTGGATGCTAACAGCCACCAGTACTTC | AAGATCCATGTTGGTCTT |
| rs849540 | ACGTTGGATGCAAGAGCTCAGGAGAGAAAC | ACGTTGGATGAAATGCAGCAGTGCATTGAC | gACAAAGGAGCCAGAAG |
| rs9287421 | ACGTTGGATGTGTACGAATCAGAGAGACAG | ACGTTGGATGTATTTTGGGAGGAGAGCCAG | GAGACAGTATATGGACAGC |
| rs9474972 | ACGTTGGATGCTGGTAATAAGTTGACCCAC | ACGTTGGATGGGACAGTGAAGGTAGAAGTG | CAACATATTAACAGCAATATCTAC |
| rs951260 | ACGTTGGATGAATCAACCACAGGAACTCAG | ACGTTGGATGTCTGGCTCTTTCACCCAAAC | gCAGGAACTCAGGAATGG |
| rs990157 | ACGTTGGATGTTGGCATCCATACTGCTGTG | ACGTTGGATGTGTGACATGTAAGGGGATTG | TGCTGTGAATAGCTGTAATG |
| SNP_A-1782550^#^ | ACGTTGGATGTAAAGCTACATGGCACCATC | ACGTTGGATGTGATGGCTTAGCTTGGGCTC | cacGGCACCATCAGCTCCA |

# Affymetrix 500k probe set ID, rs number not available.
