# Supplementary material for: Genome-Wide Scan on Total Serum IgE Levels Identifies FCER1A as Novel Susceptibility Locus
Source: PLoS Genet. 2008 Aug 22;4(8):e1000166. doi: 10.1371/journal.pgen.1000166 (PMC2565692; doi:10.1371/journal.pgen.1000166)
Supplement: Table S5 — Primers used to amplify the exons of FCER1A. (0.04 MB DOC) [file pgen.1000166.s007.doc]

|  | **Forward** | **Reverse** |
| --- | --- | --- |
| **Exon 1** | GCAGTAGCCTCCCTTAATGAC | CCTACTCTCAAGGAAGGGGC |
| **Exon 2** | AGAAGCAAAACCAGGCACAG | CAGCTGCTCCCCAAATTC |
| **Exon 3** | CCCACACCCAGATTCTAGTCC | AGGAAAGCAATGCCCAGAC |
| **Exon 4** | TGATTGTCAGAATATTGCTTCG | TCTGGTGTCCTAACCCTTGG |
| **Exon 5** | GACACATGCTCTATGCGTGG | AGCTGCAACTGCTCAGGC |
| **Exon 6** | CAAAGCTTGGTCTTTCTCTTAGG | AGAACTTCATGGTCCTTGCC |
