# Supplementary material for: Genome-Wide Scan on Total Serum IgE Levels Identifies FCER1A as Novel Susceptibility Locus
Source: PLoS Genet. 2008 Aug 22;4(8):e1000166. doi: 10.1371/journal.pgen.1000166 (PMC2565692; doi:10.1371/journal.pgen.1000166)
Supplement: Table S6 — Mutational analysis of FCER1A exons. (0.04 MB DOC) [file pgen.1000166.s008.doc]

| **SNP** | **Sequence variant** | **Position** | **Gene structure** | **MAF** | **Analyzed (n)** |
| --- | --- | --- | --- | --- | --- |
| rs2251746 | T/C | chr1:157538684 | intronic | 0.3 | 91 |
| Mutation 1 | A/G | chr1:157542690 | intronic | 0.005 | 79 |
| rs41264475 | C/A | chr1:157544313 | exon 6 | 0.03 | 95 |
| Mutation 2 | A/G | chr1:157544428 | exon 6 (3' UTR) | 0.005 | 95 |
| rs7549785 | G/A | chr1:157544492 | exon 6 (3’ UTR) | 0.17 | 95 |
