# Supplementary material for: Genome-Wide Scan on Total Serum IgE Levels Identifies FCER1A as Novel Susceptibility Locus
Source: PLoS Genet. 2008 Aug 22;4(8):e1000166. doi: 10.1371/journal.pgen.1000166 (PMC2565692; doi:10.1371/journal.pgen.1000166)
Supplement: Table S7 — Associations between FCERA1 haplotypes and IgE levels in KORA S4. Results correspond to the single SNP analyses where presence of A (rs2427837) and C (rs2251746) alleles at respective positions were strongly associated. (0.05 MB DOC) [file pgen.1000166.s009.doc]

| **No** | **rs2494262** | **rs2427837** | **rs12565775** | **rs2427824** | **rs3845625** | **rs2427827** | **rs2251746** | **freq** | **95% CI** | | **est** | **95%CI** | | **p-value** |
| --- | --- | --- | --- | --- | --- | --- | --- | --- | --- | --- | --- | --- | --- | --- |
| 1 | A | **A** | A | C | C | C | **C** | 0.2637 | 0.2541 | 0.2732 | -0.225 | -0.312 | -0.138 | 4.1x10^-7^ |
| 2 | A | G | A | C | C | C | T | 0.1838 | 0.1754 | 0.1922 | -0.045 | -0.141 | 0.051 | 0.353 |
| 3 | A | G | C | C | C | C | T | 0.0508 | 0.0461 | 0.0556 | 0.090 | -0.063 | 0.243 | 0.247 |
| 4 | C | G | A | C | T | T | T | 0.1627 | 0.1547 | 0.1707 | 0.008 | -0.090 | 0.106 | 0.869 |
| 5 | C | G | C | C | C | C | T | 0.0474 | 0.0428 | 0.0520 | 0.010 | -0.145 | 0.165 | 0.901 |
| 6 | * | * | * | * | * | * | * | 0.0239 | 0.0206 | 0.0272 | 0.041 | -0.199 | 0.281 | 0.739 |
| 7 | C | G | A | T | C | T | T | 0.2676 | 0.2580 | 0.2773 |  |  |  |  |

* haplotype frequency < 0.01.
