# Supplementary material for: Genome-Wide Scan on Total Serum IgE Levels Identifies FCER1A as Novel Susceptibility Locus
Source: PLoS Genet. 2008 Aug 22;4(8):e1000166. doi: 10.1371/journal.pgen.1000166 (PMC2565692; doi:10.1371/journal.pgen.1000166)
Supplement: Table S8 — Association analysis of FCERA1 and RAD50 variants with AE in 562 German AE trios and with asthma in 638 UK asthma cases and 633 controls. (0.06 MB DOC) [file pgen.1000166.s010.doc]

|  |  |  | **German families** | | **UK case-control** | |
| --- | --- | --- | --- | --- | --- | --- |
|  |  |  | **Atopic eczema** | **Total IgE** | **Asthma** | **Total IgE** |
| **SNP** | **Gene** | **Position** | **P-value** | **P-value** | **P-value** | **P-value** |
| rs2511211 | FCER1A | chr1:157497095 | 1.00 | 0.80 | 0.16 | 0.017 |
| rs10489854 | FCER1A | chr1:157515483 | 0.34 | 0.46 | 0.58 | 0.90 |
| rs2494262 | FCER1A | chr1:157520296 | 0.12 | 0.45 | 0.56 | 0.029 |
| rs2427837 | FCER1A | chr1:157525169 | 0.88 | 0.21 | 0.58 | 0.005 |
| rs12565775 | FCER1A | chr1:157528779 | 0.39 | 0.97 | 0.91 | 0.82 |
| rs2427824 | FCER1A | chr1:157531686 | 0.55 | 0.63 | 0.80 | 0.33 |
| rs3845625 | FCER1A | chr1:157532500 | 0.32 | 0.83 | 0.34 | 0.05 |
| rs2427827 | FCER1A | chr1:157538435 | 0.07 | 0.66 | n.a. | n.a. |
| rs2251746 | FCER1A | chr1:157538684 | 0.81 | 0.08 | 0.54 | 0.004 |
| rs6884762 | RAD50 | chr5:131966629 | 0.19 | 0.68 | 0.47 | 0.043 |
| rs17772565 | RAD50 | chr5:131980304 | 0.52 | 0.07 | 0.14 | 0.38 |
| rs17772583 | RAD50 | chr5:131981409 | 0.00962 | 0.69 | 0.82 | 0.12 |
| rs3798135 | RAD50 | chr5:131993008 | 0.00743 | 0.90 | 0.017 | 0.008 |
| rs2240032 | RAD50 | chr5:132005026 | 0.00805 | 0.82 | 0.002 | 0.002 |
