# Supplementary material for: Genome-Wide Scan on Total Serum IgE Levels Identifies FCER1A as Novel Susceptibility Locus
Source: PLoS Genet. 2008 Aug 22;4(8):e1000166. doi: 10.1371/journal.pgen.1000166 (PMC2565692; doi:10.1371/journal.pgen.1000166)
Supplement: Table S10 — Genes that have been associated with total IgE ordered by their chromosomal position. (0.16 MB DOC) [file pgen.1000166.s012.doc]

| **Gene** | **Chromosomal location**^‡^ | **SNPs covered by the Affymetrix screening panel (gene +/- 100kb)** | **Significant variation reported**^§^ | **Affymetrix SNP(s) in LD (r^2^>0,6) with the reported variant** | **Estimate** | **P-value** | **Reference^#^** |
| --- | --- | --- | --- | --- | --- | --- | --- |
| ***FLG*** | 1q21 | rs7345, rs10082235, rs3891075, rs7541193, rs6427005, rs6698058, rs9426902, rs4073768, rs6682411, rs11583896, rs16835600, rs4434872, rs4567311, rs4341393, rs4363451, rs9427232, rs12129468, rs4845576, rs4520447, rs4845578, rs11585416, rs12142130 | R501X^$^ | - | - | - | [8,14,15,16] |
|  |  |  | 2282del4^$^ | - | - | - |  |
| ***CTLA4*** | 2q33 | rs10193976, rs10490573, rs11571292, rs11571293, rs11571304, rs11689629, rs12467195, rs16840016, rs17533594, rs17533727, rs1980422, rs231747, rs231790, rs231797, rs231814, rs3115966, rs3116487, rs3116497, rs3181106, rs3181107, rs3769683, rs4673259, rs4675367 | rs231775 | rs11571292 | -0.0013 | 0.9793 | [17,18] |
|  |  |  | (AT)_n_ 3’UTR^$^  MH30/C^$^  CT60/A^$^  JO31/T^$^  JO30/A^$^  JO27_1/C^$^ | -  -  -  -  -  - | -  -  -  -  -  - | -  -  -  -  -  - |  |
| ***IRF1*** | 5q31 | rs274561, rs7731390, rs274554, rs274552, rs274547, rs6596075, rs17771891, rs4705943, rs4705944, rs2285673, rs11242111, rs6874639, rs11744116, rs4540166, rs4371745, rs10059611, rs10077785, rs2522057, rs10072700, rs2548999, rs2548997, rs739719, rs739718, rs2069812, rs2706347 | rs17622656 | rs2522057 | -0.0851 | 0.0932 | [19] |
| ***IL13*** | 5q31 | rs3798135, rs2040704, rs7737470, rs2158177, rs20541, rs2069757, rs2243218, rs2243248 | rs20541 | rs20541 | 0.2040 | 0.0005 | [20,21,22,23,24] |
|  |  |  | rs1800925 | - | - | - |  |
| ***IL4*** | 5q31 | rs2227284, rs2227282, rs2243290, rs2023822  rs11242127, rs194395, rs402959, rs30527, rs2069757, rs2243218, rs2243248 | rs2243250 | rs2243290  rs194395  rs402959 | 0.1354  0.1244  0.1323 | 0.0552  0.0993  0.0785 | [25,26] |
|  |  |  | rs2070874 | rs2243290  rs194395  rs402959 | 0.1354  0.1244  0.1323 | 0.0552  0.0993  0.0785 |  |
|  |  |  | rs2227284 | rs2227284  rs2227282  rs11242127 | 0.0635  0.0658  0.0642 | 0.2594  0.2389  0.2474 |  |
|  |  |  | rs2243266 | rs2243290  rs194395  rs402959  rs30527 | 0,1354  0,1244  0,1323  0,1432 | 0,0552  0,0993  0,0785  0,0571 |  |
|  |  |  | rs2243274 | rs2243290  rs194395  rs402959  rs30527 | 0,1354  0,1244  0,1323  0,1432 | 0,0552  0,0993  0,0785  0,0571 |  |
|  |  |  | rs2243288 | rs2243290  rs194395  rs402959  rs30527 | 0,1354  0,1244  0,1323  0,1432 | 0,0552  0,0993  0,0785  0,0571 |  |
|  |  |  | rs2243289 | rs2243290  rs194395  rs402959  rs30527 | 0,1354  0,1244  0,1323  0,1432 | 0,0552  0,0993  0,0785  0,0571 |  |
|  |  |  | rs2243290 | rs2243290  rs194395  rs402959  rs30527 | 0,1354  0,1244  0,1323  0,1432 | 0,0552  0,0993  0,0785  0,0571 |  |
|  |  |  | rs2243284 | - | - | - |  |
| ***CD14*** | 5q31 | rs1835959, rs250432, rs250430, rs250428, rs2282803, rs6550, rs2253378, rs2569169, rs2163786, rs2569192, rs5744441, rs3822356, rs17208187, rs753279, rs801399, rs2563335, rs801186, rs702396, rs801168, rs801167, rs17286731, rs801183 | rs2569190 | rs2163786  rs753279  rs801399  rs2563335  rs801168  rs801167  rs801183 | -0.0111  0.0160  -0.0442  -0.0367  -0.0350  -0.0361  -0.0391 | 0.8236  0.7477  0.3718  0.4593  0.4815  0.4672  0.4298 | [27,28] |
| ***SPINK5*** | 5q32 | rs1363530, rs17538716, rs7707803, rs10463395, rs1422998, rs1422997, rs6895394, rs17641748,  rs10491342, rs7713918, rs7727019, rs17107665,  rs1363720, rs1363719, rs953310, rs11958071,  rs17718403, rs17718420, rs3756688, rs1423007,  rs2287774, rs9325064, rs986494, rs1423001, rs12108690, rs4529181, rs2303066, rs2303069, rs1422987, rs3777142, rs988885, rs3815738, rs1862439, rs3777138, rs2052531, rs9325073, rs17704908, rs2112767, rs3756690, rs12187820, rs7717500, rs10068988, rs10067334, rs6881658, rs6895745, rs7703761, SNP_A-1807295, rs4270684, rs4452547, rs4259160, rs4499819, rs11745313, rs11168039, rs4705232, rs4705233, rs17096690, rs12517179, rs1432693, rs986673 | rs2303067 | rs10463395  rs1422998  rs1422997  rs6895394  rs7713918  rs3756688  rs1423007  rs9325064  rs986494  rs1423001  rs12108690  rs4529181  rs2303066  rs988885  rs1862439 | 0.0854  0.0857  0.1049  0.1206  0.1136  0.1038  0.0811  0.0923  0.0593  0.0908  0.0890  0.0799  0.0611  0.0664  0.0653 | 0.0867  0.0879  0.0416  0.0172  0.0239  0.0568  0.1119  0.0690  0.2597  0.0736  0.0796  0.1123  0.2067  0.1908  0.1982 | [29] |
| ***ADRB2*** | 5q31-32 | rs17108682, rs13176278, rs9325115, rs2400642  rs1363543, rs6885118, rs1465405, rs6867058, rs6885216, rs919724, rs2082398, rs30309, rs30313, rs30319, rs30325, rs246503, rs246502, rs9285673, rs1347110, rs877741, rs877743, rs11168066, rs11959615, rs17778257, rs2400707, rs11957757, rs10066266, rs6897873, rs11168071, rs6580586, rs6884617, rs1432628, rs17640705, rs1560641, rs12520348, rs10042578, rs10036926, rs4705283, rs4705284, rs4705285, rs10875641, rs10075995, rs11746220, rs11744605, rs17653203, rs17461431, rs4705291, rs4705292, rs11740851, rs12189287 | rs1042714 | rs11168066  rs11959615  rs2400707 | -0.0733  -0.0775  -0.0607 | 0.1349  0.1356  0.2249 | [30,31] |
| ***NOD1*** | 7p15 | rs38437, rs38449, rs38452, rs38466, rs38474, rs38488, rs2529439, rs11761080, rs12532057, rs16875290, rs2267710, rs975537, rs973002, rs929377, rs2240404, rs2190242, rs1003929, rs4723002, rs4723003, rs255121, rs255131, rs255152, rs255155, rs255156, rs255166, rs11771217, rs1971677, rs2075005, rs1061644, rs7810199 | rs2907748 | - | - | - | [32,33] |
|  |  |  | rs2907749 | - | - | - |  |
|  |  |  | rs2075822 | - | - | - |  |
|  |  |  | rs2975632 | - | - | - |  |
|  |  |  | ND1 +32656^+^  (rs6958571) | - | - | - |  |
|  |  |  | rs2736726 | - | - | - |  |
|  |  |  | rs2075817 | - | - | - |  |
|  |  |  | rs3020207 | - | - | - |  |
|  |  |  | rs2075818 | - | - | - |  |
|  |  |  | rs2235099 | - | - | - |  |
|  |  |  | rs5743368 | - | - | - |  |
| ***GPRA*** | 7p15-14 | rs17198982, rs17788843, rs324978, rs324981, rs1419780, rs325464, rs17199659, rs17789420, rs17789642, rs17200135, rs17170015, rs17200455, rs10263313, rs11768189, rs17790961, rs4723388, rs2057819, rs2557768, rs1649218, rs1637670, rs1649238, rs1637682, rs10267957, rs328906, rs2023328, rs328928, rs2392303, rs329234, rs329235, rs329240, rs329251, rs329271,  rs329277, rs329559, rs9648411, rs17158356 | SNP546333^$^ | - | - | - | [34,35] |
|  |  |  | rs740347 | - | - | - |  |
| ***IL18*** | 11q22 | rs10891323, rs578784, rs243908, rs544354, rs360729, rs1834481, rs360722, rs7106524, rs360718, rs10891337, rs11214127, rs4935984, rs11214136, rs2518345, rs3819331, rs2564873, rs2518360, rs17490906 | rs360718 | rs360718  rs10891337  rs11214127 | -0.0441  -0.0137  -0.0149 | 0.4286  0.8105  0.7934 | [36,37] |
|  |  |  | rs360717 | rs360718  rs10891337  rs11214127 | -0.0441  -0.0137  -0.0149 | 0.4286  0.8105  0.7934 |  |
|  |  |  | rs360721 | - | - | - |  |
|  |  |  | rs1946519 | - | - | - |  |
|  |  |  | rs187238 | - | - | - |  |
| ***STAT6*** | 12q13 | rs17119327, rs17119330, rs17119344, rs1552245, rs697222, rs17119386, rs2270737, rs7298475, rs17119416, rs12368672, rs4759277, rs1466535, rs7975818, rs1800176, rs4074308, rs1800168 | rs3024974 | rs17119327  rs17119330  rs17119344  rs1552245  rs697222  rs17119386  rs2270737  rs7298475  rs17119416 | -0.0446  -0.0249  -0.0590  -0.0590  -0.1693  -0.0509  -0.0707  -0.0734  -0.0473 | 0.6214  0.7769  0.5134  0.5134  0.0288  0.5743  0.4350  0.4195  0.5911 | [38,39,40] |
|  |  |  | rs324011 |  |  |  |  |
| ***VDR*** | 12q13 | rs2072115, rs10747521, rs12818241, rs2544025, rs3782908, rs4760636, rs7418, rs2544029, rs2525051, rs7299689, rs7971594, rs7965281, rs10783215, rs2525046, rs7962898, rs11168268, rs2248098, rs2239182, rs2239180, rs12717991, rs2189480, rs2254210, rs11574046, rs2238136, rs4760658, rs4516035, rs11568820, rs7310552, rs7132324, rs10875700, rs7311856, rs4760671, rs7972027, rs11168314, rs11168316, rs10875704, rs10875705, rs10747527, rs10783221, rs11168325, rs11168326, rs1541408, rs2286024, rs2071358, rs1034762, rs7971880 | rs2239185 | - | - | - | [41,42] |
|  |  |  | rs731236 | rs2239182 | -0.0331 | 0.5072 |  |
|  |  |  | rs7975232 | rs7965281  rs10783215  rs2525046  rs7962898  rs11168268  rs2248098 | 0.0182  0.0193  0.0303  0.0545  0.0364  0.0449 | 0.7130  0.6997  0.5426  0.2618  0.4657  0.3612 |  |
|  |  |  | rs2239179 | rs2239182 | -0.0331 | 0.5072 |  |
|  |  |  | rs1540339 | rs12717991 | 0.0544 | 0.2668 |  |
| ***NOS1*** | 12q24 | rs7967489, rs7957302, rs957363, SNP_A-2052092, rs11068363, rs10850780, rs4238052, rs11068384, rs10774905, rs9658536, rs904658, rs9658498, rs3741476, rs10774909, rs2650163, rs12829185, rs816358, rs6490121, rs1607817, rs3825102, rs884847, rs7309163, rs532967, rs733334, rs7961147, rs478597, rs1483757, rs816293, rs471871, rs17509231, rs9658253, rs816292, rs12307921, rs492623, rs12826308, rs1552229, rs1552230, rs1384873, rs10850820, rs7971155, rs11068469, rs10774915, SNP_A-1804024, rs708849, rs10850824, rs10774923, rs10850828, rs11068499, rs11068501, rs10774926, rs1093297, rs11068503, rs11068505, rs1068944, rs1580855, rs7298492, rs10850833 | rs2682826 | rs10774909  rs12829185 | 0.0505  -0.0320 | 0.4174  0.6049 | [43,44] |
| ***PHF11*** | 13q14 | rs7338471, rs17072869, rs11617824, rs9535233, rs2057412, rs11619997, rs9591258, rs17072878, rs4942830, rs7992603, rs12429883, rs1980795, rs2031532, rs9568232, rs2981, rs3829366, rs6561534, rs9568239, rs3186024, rs7995684, rs1046028, rs3186013, rs7995006, rs3794376, rs1536192, rs7333969, rs3751383, rs17069328, rs9596148, rs9568258, rs9596150, rs9562900, rs11148150, rs11148151, rs882970, rs7981396, rs9526580, rs4941650, rs7327293, rs7331252, rs7332029, rs7335220 | b5_2^$^  b4_2^$^  b5_3^$^ | - | - | - | [45] |
| ***IL4RA-IL21R cluster*** | 16p12-11 | rs11641348, rs7186151, rs4547335, rs8059713, rs4395078, rs4547336, rs8057585, rs8044444, rs6498012, rs3024547, rs2239349, rs3024585, rs3024613, rs3024614, rs3024668, rs3024676, rs8832, rs16976728, rs4787426, rs7191188, rs6498015, rs6498016, rs2040788, rs6498017, rs722516, rs722517, rs1859308, rs7205704, rs8060368, rs2189521, rs11074858, rs7199138, rs8057551, rs8061992, rs3093315, rs3093317, rs3093341, rs3093366, rs3093367, rs3093378, rs12445873, rs12925626, rs232075, rs232081, rs232086, rs7198785, rs4787967 | rs1805011 | rs3024676 | -0.0377 | 0.5731 | [46,47,48] |
|  |  |  | rs1805013 | - | - | - |  |
|  |  |  | rs1801275 | rs3024676 | -0.0377 | 0.5731 |  |
|  |  |  | rs1805012 | rs3024676 | -0.0377 | 0.5731 |  |
|  |  |  |  |  |  |  |  |
| ***CARD15*** | 16q21 | rs933566, rs8047222, rs745230, rs7199150, rs7186163, rs2066849, rs8050932, rs7202124, rs9933594, rs4785448, rs5743263, rs8057341, rs17221417, rs17312836, rs2066843, rs748855, rs1861758, rs1077861, rs3135499, rs8060598, rs3785142, rs4785450, rs17224078, rs1861760, rs4785460, rs6500336, rs9922324, rs16948899 | rs2066845 | - | - | - | [49,50] |
|  |  |  | 3020insC^$^ | - | - | - |  |

^‡^ location based on NCBI 36 entrez gene (http://www.ncbi.nlm.nih.gov/sites/entrez).

§ reported variant associated with total IgE.

# references reporting association with total IgE (only positive association reports listed).

+ complex insertion–deletion polymorphism (indel), partially identified as rs6958571.

$ no rs-number available.

Abbreviations: ADRB2 = beta-2-adrenergic receptor; CARD15 = nucleotide-binding oligomerization domain protein 2 (= NOD2); CD14 = monocyte differentiation antigen CD14; CTLA4 = cytytoxic T lymphocyte-associated 4; FLG = filaggrin; GPRA = G protein-coupled receptor 154; IL4 = interleukin 4; IL4RA = interleukin 4 receptor, alpha; IL13 = interleukin 13; IL18 = interleukin 18; IL21R = interleukin 21 receptor; NOD1 = caspase recruitment domain-containing protein 4 (= CARD4); NOS1 = nitirc oxide synthase 1; PHF11 = PHD finger protein 11; SPINK5 = serine protease inhibitor, Kazal-type, 5; STAT6 = signal transducer and activator of transcription 6; VDR = vitamin D receptor.

## We searched public databases for published candidate gene association studies for total IgE. We list only those for which association with total IgE has been reported in at least two independent white study populations of sufficient size (>200 subjects). In some of the cases we were not able to derive rs numbers. LD calculations are based on HapMap (HapMap Data Release 23a/PhaseII Mar08, on NCBI B36 assembly, dbSNP b126; [***http://www.hapmap.org/index.html.en***](http://www.hapmap.org/index.html.en)). References refer to positive reports only.
