# Supplementary material for: Genome-Wide Scan on Total Serum IgE Levels Identifies FCER1A as Novel Susceptibility Locus
Source: PLoS Genet. 2008 Aug 22;4(8):e1000166. doi: 10.1371/journal.pgen.1000166 (PMC2565692; doi:10.1371/journal.pgen.1000166)
Supplement: Table S11 — Affymetrix SNPs in selected candidate genes for total IgE, which yielded a nominal p-value <0.05 in the GWAS. Genes are ordered by their chromosomal position. (0.14 MB DOC) [file pgen.1000166.s013.doc]

| **Gene** | **Chromosomal location** | **SNPs covered by the Affymetrix screening panel (+/- 100kb)** | **Position** | **Estimate** | **P-value** |
| --- | --- | --- | --- | --- | --- |
| *SELP* | 1q22-25 | rs7474070 | 167782247 | -0.3007 | 0.0047 |
|  |  | rs10910879 | 167784811 | 0.1516 | 0.0209 |
|  |  | rs12404218 | 167790915 | -0.2945 | 0.0054 |
|  |  | rs742058 | 167823078 | -0.2670 | 0.0416 |
|  |  | rs1157459 | 167835109 | -0.2845 | 0.0301 |
|  |  | rs7882773 | 167835265 | -0.1532 | 0.0100 |
|  |  | rs2281006 | 167842527 | 0.1279 | 0.0342 |
|  |  | rs17565766 | 167847788 | 0.1272 | 0.0421 |
|  |  | rs17565793 | 167847925 | 0.1232 | 0.0492 |
|  |  | rs7063044 | 167848418 | 0.1265 | 0.0447 |
|  |  | rs6608452 | 167848771 | 0.1276 | 0.0422 |
|  |  | rs12720462 | 167949349 | -0.1742 | 0.0400 |
| *DPP10* | 2q14 | rs11123288 | 115680236 | -0.1278 | 0.0293 |
|  |  | rs7568150 | 116139130 | -0.1107 | 0.0452 |
|  |  | rs10184097 | 116162717 | -0.1086 | 0.0475 |
|  |  | rs4849421 | 116189249 | -0.1164 | 0.0350 |
|  |  | rs1374271 | 116251152 | 0.1385 | 0.0123 |
|  |  | rs6735355 | 116264756 | -0.1424 | 0.0179 |
|  |  | rs3768692 | 116318073 | -0.1584 | 0.0407 |
|  |  | rs7420865 | 116330344 | -0.1721 | 0.0265 |
|  |  | rs272041 | 116345018 | 0.1261 | 0.0182 |
| *IRF1* | 5q31 | rs274561 | 131747127 | 0.1109 | 0.0269 |
|  |  | rs2706347 | 131933016 | 0.2357 | 4.1x10^-5^ |
| *IL13* | 5q31 | rs3798135 | 131993008 | 0.2271 | 0.0001 |
|  |  | rs2040704 | 132001076 | 0.2210 | 0.0001 |
|  |  | rs7737470 | 132001962 | 0.2312 | 4.8x10^-5^ |
|  |  | rs2158177 | 132011957 | 0.1834 | 0.0020 |
|  |  | rs20541 | 132023863 | 0.2040 | 0.0005 |
| *SPINK5* | 5q32 | rs7707803 | 147368427 | -0.1640 | 0.0234 |
|  |  | rs1422997 | 147373857 | 0.1049 | 0.0416 |
|  |  | rs6895394 | 147379211 | 0.1206 | 0.0172 |
|  |  | rs7713918 | 147393844 | 0.1136 | 0.0239 |
| *GPRA* | 7p15-14 | rs324978 | 34587572 | -0.1084 | 0.0472 |
|  |  | rs17200455 | 34651236 | -0.1840 | 0.0033 |
|  |  | rs10263313 | 34654233 | -0.1808 | 0.0040 |
|  |  | rs4723388 | 34716060 | 0.1647 | 0.0142 |
|  |  | rs329271 | 34849117 | 0.1656 | 0.0136 |
| *FCER1B* | 11q13 | rs1286289 | 59583253 | -0.1195 | 0.0258 |
|  |  | rs1286170 | 59588506 | -0.1192 | 0.0231 |
|  |  | rs501697 | 59589699 | -0.1276 | 0.0182 |
|  |  | rs528823 | 59593673 | -0.1253 | 0.0205 |
|  |  | rs558788 | 59608654 | -0.1196 | 0.0269 |
|  |  | rs558678 | 59608689 | -0.1271 | 0.0202 |
| *STAT6* | 12q13 | rs697222 | 55721389 | -0.1693 | 0.0288 |
|  |  | rs12368672 | 55798737 | 0.1673 | 0.0009 |
|  |  | rs4759277 | 55819957 | 0.1464 | 0.0041 |
|  |  | rs1466535 | 55820737 | 0.1475 | 0.0041 |
| *NOS1* | 12q24 | rs10850820 | 116293199 | 0.0986 | 0.0452 |
|  |  | rs10774923 | 116343062 | 0.1076 | 0.0267 |
|  |  | rs11068501 | 116357854 | 0.1377 | 0.0449 |
|  |  | rs11068503 | 116364878 | 0.0993 | 0.0452 |
| *IL4RA-IL21R* Cluster | 16p12-11 | rs7186151 | 27156882 | 0.1200 | 0.0222 |
|  |  | rs3024613 | 27271754 | 0.1031 | 0.0382 |
|  |  | rs16976728 | 27289213 | 0.1059 | 0.0410 |
|  |  | rs7205704 | 27308394 | 0.1550 | 0.0056 |
|  |  | rs3093378 | 27365559 | 0.1156 | 0.0269 |
|  |  | rs12445873 | 27381715 | 0.1685 | 0.0117 |
|  |  | rs12925626 | 27384049 | 0.1676 | 0.0121 |
|  |  | rs232075 | 27423252 | 0.1701 | 0.0112 |
|  |  | rs232081 | 27436681 | 0.1596 | 0.0204 |
|  |  | rs7198785 | 27465250 | 0.1953 | 0.0032 |
|  |  | rs4787967 | 27466060 | 0.1971 | 0.0031 |
| *CARD15* | 16q21 | rs9922324 | 49423561 | 0.2370 | 0.0364 |
| *ADAM33* | 20p13 | rs464831 | 3501750 | 0.1319 | 0.0172 |
|  |  | rs235544 | 3508655 | 0.1253 | 0.0357 |
