# Supplementary material for: Genome-Wide Scan on Total Serum IgE Levels Identifies FCER1A as Novel Susceptibility Locus
Source: PLoS Genet. 2008 Aug 22;4(8):e1000166. doi: 10.1371/journal.pgen.1000166 (PMC2565692; doi:10.1371/journal.pgen.1000166)
Supplement: Text S1 — Supplementary information. (0.10 MB DOC) [file pgen.1000166.s014.doc]

**Genome-wide scan on total serum IgE levels identifies**

***FCER1A* as susceptibility locus**

Stephan Weidinger^1,2,17^, Christian Gieger^3,4,17^, Elke Rodriguez^2^, Hansjörg Baurecht^2,5^, Martin Mempel^1,2^, Norman Klopp^3^, Henning Gohlke^3^, ,Stefan Wagenpfeil^5,6^, Markus Ollert^1,2^, Johannes Ring^1^, Heidrun Behrendt^2^, Joachim Heinrich^3^, Natalija Novak^7^, Thomas Bieber^7^, Ursula Krämer^8^, Dietrich Berdel^9^, Andrea von Berg^9^, Carl Peter Bauer^10^, Olf Herbarth^11^, Sibylle Koletzko^12^, Holger Prokisch^13,14^, Divya Mehta^13,14^, Thomas Meitinger^13,14^, Martin Depner^12^, Erika von Mutius^12^, Liming Liang^15^, Miriam Moffatt^16^, William Cookson^16^, Michael Kabesch^12^, H.-Erich Wichmann^3,4^, Thomas Illig^3^

^1^ Department of Dermatology and Allergy, Technische Universität München, München, Germany

^2^ Division of Environmental Dermatology and Allergy, Helmholtz Zentrum München, Neuherberg and ZAUM-Center for Allergy and Environment, Technische Universität München, München, Germany

^3^ Institute of Epidemiology, Helmholtz Zentrum München, German Research Center for Environmental Health, Neuherberg, Germany

^4^ Institute of Medical Informatics, Biometry and Epidemiology, Ludwig-Maximilians-Universität München, München, Germany

^5^ IMSE Institute for Medical Statistics and Epidemiology, Technische Universität München, München, Germany

^6^ Graduate School of Information Science in Health (GSISH), Technische Universität München, München, Germany

^7^ Department of Dermatology and Allergy, University of Bonn, Bonn, Germany

^8^ IUF -Institut für Umweltmedizinische Forschung at the Heinrich-Heine-University, Düsseldorf, Germany

^9^ Marien-Hospital, Wesel, Germany

^10^ Department of Pediatrics, Technische Universität München, München, Germany.

^11^ Department of Human Exposure Research and Epidemiology, UFZ-Centre for Environmental Research Leipzig, Leipzig, Germany

^12^ University Children’s Hospital, Ludwig-Maximilians-Universität München, München, Germany

^13^ Institute of Human Genetics, Helmholtz Zentrum München, German Research Center for Environmental Health, Neuherberg, Germany

^14^ Institute of Human Genetics, Klinikum rechts der Isar, Technische Universität München, München, Germany.

^15^ Center for Statistical Genetics, Department of Biostatistics, School of Public Health, Ann Arbor, Michigan, USA.

^16^ National Heart and Lung Institute, Imperial College London, London, UK.

^17^ These authors contributed equally to this work.

**Supplementary Information**

**Supplementary Methods** 3

Subjects and study design *3*

KORA S3/F3 500K genotyping and quality control *3*

Detailed information on SNP selection for replication and finemapping *4*

SNP genotyping and quality control in the replication samples *4*

Mutational analysis *FCER1A 5*

Haplotype analysis *5*

Analysis of population sub-structure *6*

Extended SNP analysis in the *RAD50*-*IL13* region in a subset of 526 children from the ISAAC replication cohort *6*

Additional association analysis of *FCER1A* and *RAD50* with AE and asthma *6*

Analysis of previously reported candidate genes *7*

# Supplementary Methods

## Subjects and study design

A description of the GWA study population and the replication samples is given in Table S1.

## KORA S3/F3 500K genotyping and quality control

Genotyping for KORA S3/F3 500K was performed using Affymetrix 500K Array Set consisting of two chips (Sty I and Nsp I). Hybridisation of genomic DNA was done in accordance with the manufacturer’s standard recommendations. Genotypes were determined using BRLMM clustering algorithm (http://www.affymetrix.com/support/technical/whitepapers/brlmm_ whitepaper.pdf). The genotypes were determined in batches of at least 400 chips. For quality control purposes, we applied a positive control and a negative control DNA every 48 samples. The overall genotyping efficiency of the GWA was 98.26 %. Before statistical analysis, we performed filtering of both conspicuous chips and SNPs based on quality measures to ensure robustness of association analysis. On chip level only subjects with overall genotyping efficiencies of at least 93% for both chips and at most one discordant call for 50 SNPs being on both chips were included. In addition the called gender has to agree with the gender in the KORA study database. On SNP level from a total of 500,568 SNPs, we excluded for the purpose of this analysis all SNPs on chromosome X leaving 490,032 autosomal SNPs for the GWA screening step. From these 353,569 (72.15%) SNPs passed all quality control criteria, and were selected for the subsequent association analyses. Criteria leading to exclusion were genotyping efficiency < 95% and minor allele frequency (MAF) < 3%. An exact Fisher test has been used to detect deviations from Hardy Weinberg Equilibrium, and we excluded all SNPs with P-values below 10^-6^. Details on the number of excluded SNPs per quality criterion are described in Table S2.

## Detailed information on SNP selection for replication and finemapping

For replication in KORA S4 (population-based, n=3,890), we used the following inclusion criteria:

1. *P* < 10^-4^ in stage 1 analysis
2. *P* <10^-3^ with at least one neighboring SNP (+/-100 kb) with *P*<10^-3^

Details on the SNPs selected for replication are shown in Table S3.

After we had identified the *FCER1A* and *RAD50* region through the genome-wide scan and replication, we additionally analysed 6 tagging SNPs in *FCER1A* as well as 4 tagging SNPs in *RAD50* using the pairwise tagging algorithm (r^2^ >0.8) implemented in HAPLOVIEW 3.3 (HapMap data release #22, March 2007, on NCBI B36 assembly, dbSNP b126. In addition, two previously described functional SNPs of *FCER1A* [1,2] and 2 SNPs in intron 24 of the *RAD50* hypersensitive site 7 (RHS7) [3] were genotyped.

## SNP genotyping and quality control in the replication samples

Genotyping of SNPs was performed with the iPLEX™ (Sequenom San Diego, CA, USA) method by means of matrix assisted laser desorption ionisation-time of flight mass spectrometry method (MALDI-TOF MS, Mass Array™, Sequenom, San Diego, CA, USA) according to the manufacturers instructions.(www.sequenom.com ). Shortly, 5 ng of genomic template DNA were amplified applying 5 units of HotStarTaq DNA Polymerase (Qiagen, Hilden, Germany). Thermocycling started with a single denaturation step for 15 min at 95°C, followed by 45 cycles of denaturation for 20 sec at 95°C, annealing for 30 sec at 56°C and extension at 72°C for 1 min, finally followed by 72°C for 10 min.

Subsequently PCR products were treated with shrimp alkaline phosphatase (SAP, Amersham, Freiburg, Germany) for 20 min at 37°C to remove excess dNTPs followed by 10 min at 85°C to inactivate SAP (Table S3). Base extension (iPLEX™ Sequenom, San Diego, CA) reactions in a final volume of 10 μl contained extension primers at a final concentration of 0.635 μM and 0.6 units ThermoSequenase (Amersham, Freiburg, Germany).

Base extension reaction conditions were 94°C for 2 min, followed by 40 cycles of 94°C for 5 sec, 52°C for 5 sec, and 72°C for 5 sec. Afterwards the base extension products were treated with SpectroCLEAN resin (Sequenom, San Diego, CA, USA). 20 nanoliters of the reaction solution were spotted onto a 384 format SpectroCHIP microarray (Sequenom, San Diego, CA, USA) presloaded with a matrix of 3-hydroxypicolinic acid (3-HPA). A modified Bruker Biflex MALDI-TOF MS was used for data acquisitions from the SpectroCHIP. Genotype calling was performed in real time with MassARRAY RT software version 3.0.0.4 (Sequenom, San Diego, CA, USA). For quality control 5% of the selected samples were genotyped in duplicate and in addition 4% of negative controls were used.

Primer sequences of the SNPs selected for replication and fine mapping are shown in Table S4.

## Mutational analysis FCER1A

*FCER1A* exons were amplified with intronic primers and were directly sequenced using a BigDye Cycle sequencing kit (Applied Biosystems).

Details are provided in Tables S5 and S6.

## Haplotype analysis

Haplotype reconstruction and association analysis from the unphased genotypes was performed on the KORA S4 replication sample using the SNP tagger implemented in HAPLOVIEW 3.3.2 [4] and the function *haplo.glm* implemented in the S-PLUS/R library *HaploStats* [5] (http://mayoresearch.mayo.edu/mayo/research/biostat/splusfunctions.cfm). The latter computes maximum likelihood estimates of the haplotype probabilities using an EM algorithm. All common haplotypes were included in the linear regression; the most common haplotype served as reference. Rare haplotypes (frequency <1%) were pooled and included in the model as one group. Each possible haplotype combination per individual entered the additive model as a weighted observation. The weights were calculated from the posterior probabilities of the respective pair of haplotypes. Haplotype analysis was performed on all *FCER1A* and *RAD50* SNPs replicated in KORA S4. Results of the haplotype analysis, which was computed adjusted for gender and age, are reported in Table S7.

## Analysis of population sub-structure

The association is very unlikely to be due to population stratification for two reasons: First, if the result would indeed be caused by population stratification, this stratification would have been the same in the KORA, LISA, GINI and ISAAC Study with the same substructures and the same extent of substructure in each of the three samples. Second a recent experimental assessment has found little population stratification to exist within and across Germany [6]. To test for possible population stratification in KORA S3/F3 500K an EIGENSOFT analysis was performed as described in [7]. A genomic control analysis led to a value of λ, the inflation factor, of 1.09.

## Extended SNP analysis in the RAD50-IL13 region in a subset of 526 children from the ISAAC replication cohort

Table S8 shows details of the additional SNPs typed in a subset of the ISAAC cohort and their association with total IgE. We report the p-values of a t-test in a dominant model. All tests are based on log-transformed IgE-values. Figure S2 shows the patterns of pairwise LD between the SNPs at the *RAD50-IL13* locus.

## Additional association analysis of FCER1A and RAD50 with AE and asthma

Associations of *FCER1A* and *RAD50* variants tagging the significant SNPs from the GWAS and replication studies on total IgE with the dichotomous traits AE and asthma were analysed in a cohort of 562 German parent-offspring trios for AE and a cohort of 638 asthma cases and 633 controls. German trios and UK asthma cases and controls were recruited and phenotyped as previously described [8,9]. In the family-setting we analysed association of single SNPs with eczema using the classical transmission disequilibrium test (TDT), while association with log-transformed total IgE values was analysed with the quantitative version of the TDT (QTDT). For association between asthma status and SNP genotype we fitted parametric model using the LAMP program. The program estimates disease allele frequency, a SNP allele frequency and three penetrances(disease prevalence is set to 5%) by assuming a multiplicative model and ignored linkage [10,11]. Association analysis of IgE was applied with MERLIN (-fastAssoc option). We fitted a simple regression model and used a variance component approach to account for the correlations between family members. The program estimates the additive effect of each SNP on the level of IgE and imputes missing genotype with expected genotype score using the Lander-Green algorithm [12,13].

Results are shown in Table S9.

**Post-hoc analysis of previously reported candidate genes**

In a secondary approach, we also examined the results of the GWAS with respect to previously reported candidate genes for total IgE (Table S10). To this end, we searched the public databases for candidate genes for total IgE identified in at least two independent cohorts of substantial size (>200 subjects).

We list all SNPs from within or around (±100kb) these genes covered by the affymetrix 500k screening panel. In addition, we checked for affymetrix SNP(s) in LD (r^2^>0,6) with reported variants and their association with total IgE in our GWAS. Occasionally it was not possible to pinpoint to one SNP as being more important among a number of SNPs with equally strong and potentially even independent effects (“multiple SNPs”). In addition, in some of the cases we were not able to derive rs numbers.

Table S11 shows all affymetrix SNPs from within or around (±100kb) selected candidate genes which yielded a nominal p-value < 0.05.

**References**

1. Hasegawa M, Nishiyama C, Nishiyama M, Akizawa Y, Mitsuishi K, et al. (2003) A novel -66T/C polymorphism in Fc epsilon RI alpha-chain promoter affecting the transcription activity: possible relationship to allergic diseases. J Immunol 171: 1927-1933.

2. Shikanai T, Silverman ES, Morse BW, Lilly CM, Inoue H, et al. (2002) Sequence variants in the FcepsilonRI alpha chain gene. J Appl Physiol 93: 37-41.

3. Lee GR, Spilianakis CG, Flavell RA (2005) Hypersensitive site 7 of the TH2 locus control region is essential for expressing TH2 cytokine genes and for long-range intrachromosomal interactions. Nat Immunol 6: 42-48.

4. Barrett JC, Fry B, Maller J, Daly MJ (2005) Haploview: analysis and visualization of LD and haplotype maps. Bioinformatics 21: 263-265.

5. Lake SL, Lyon H, Tantisira K, Silverman EK, Weiss ST, et al. (2003) Estimation and tests of haplotype-environment interaction when linkage phase is ambiguous. Hum Hered 55: 56-65.

6. Steffens M, Lamina C, Illig T, Bettecken T, Vogler R, et al. (2006) SNP-based analysis of genetic substructure in the German population. Hum Hered 62: 20-29.

7. Winkelmann J, Schormair B, Lichtner P, Ripke S, Xiong L, et al. (2007) Genome-wide association study of restless legs syndrome identifies common variants in three genomic regions. Nat Genet 39: 1000-1006.

8. Weidinger S, Rodriguez E, Stahl C, Wagenpfeil S, Klopp N, et al. (2007) Filaggrin mutations strongly predispose to early-onset and extrinsic atopic dermatitis. J Invest Dermatol 127: 724-726.

9. Moffatt MF, Kabesch M, Liang L, Dixon AL, Strachan D, et al. (2007) Genetic variants regulating ORMDL3 expression contribute to the risk of childhood asthma. Nature 448: 470-473.

10. Li M, Boehnke M, Abecasis GR (2005) Joint modeling of linkage and association: identifying SNPs responsible for a linkage signal. Am J Hum Genet 76: 934-949.

11. Li M, Boehnke M, Abecasis GR (2006) Efficient study designs for test of genetic association using sibship data and unrelated cases and controls. Am J Hum Genet 78: 778-792.

12. Chen WM, Abecasis GR (2007) Family-based association tests for genomewide association scans. Am J Hum Genet 81: 913-926.

13. Abecasis GR, Cherny SS, Cookson WO, Cardon LR (2002) Merlin--rapid analysis of dense genetic maps using sparse gene flow trees. Nat Genet 30: 97-101.

14. Rogers AJ, Celedon JC, Lasky-Su JA, Weiss ST, Raby BA (2007) Filaggrin mutations confer susceptibility to atopic dermatitis but not to asthma. J Allergy Clin Immunol 120: 1332-1337.

15. Weidinger S, Illig T, Baurecht H, Irvine AD, Rodriguez E, et al. (2006) Loss-of-function variations within the filaggrin gene predispose for atopic dermatitis with allergic sensitizations. J Allergy Clin Immunol 118: 214-219.

16. Novak N, Baurecht H, Schafer T, Rodriguez E, Wagenpfeil S, et al. (2008) Loss-of-function mutations in the filaggrin gene and allergic contact sensitization to nickel. J Invest Dermatol 128: 1430-1435.

17. Howard TD, Postma DS, Hawkins GA, Koppelman GH, Zheng SL, et al. (2002) Fine mapping of an IgE-controlling gene on chromosome 2q: Analysis of CTLA4 and CD28. J Allergy Clin Immunol 110: 743-751.

18. Munthe-Kaas MC, Carlsen KH, Helms PJ, Gerritsen J, Whyte M, et al. (2004) CTLA-4 polymorphisms in allergy and asthma and the TH1/ TH2 paradigm. J Allergy Clin Immunol 114: 280-287.

19. Schedel M, Pinto LA, Schaub B, Rosenstiel P, Cherkasov D, et al. (2008) IRF-1 gene variations influence IgE regulation and atopy. Am J Respir Crit Care Med 177: 613-621.

20. Liu X, Beaty TH, Deindl P, Huang SK, Lau S, et al. (2003) Associations between total serum IgE levels and the 6 potentially functional variants within the genes IL4, IL13, and IL4RA in German children: the German Multicenter Atopy Study. J Allergy Clin Immunol 112: 382-388.

21. Graves PE, Kabesch M, Halonen M, Holberg CJ, Baldini M, et al. (2000) A cluster of seven tightly linked polymorphisms in the IL-13 gene is associated with total serum IgE levels in three populations of white children. J Allergy Clin Immunol 105: 506-513.

22. Heinzmann A, Mao XQ, Akaiwa M, Kreomer RT, Gao PS, et al. (2000) Genetic variants of IL-13 signalling and human asthma and atopy. Hum Mol Genet 9: 549-559.

23. Hoffjan S, Ostrovnaja I, Nicolae D, Newman DL, Nicolae R, et al. (2004) Genetic variation in immunoregulatory pathways and atopic phenotypes in infancy. J Allergy Clin Immunol 113: 511-518.

24. Maier LM, Howson JM, Walker N, Spickett GP, Jones RW, et al. (2006) Association of IL13 with total IgE: evidence against an inverse association of atopy and diabetes. J Allergy Clin Immunol 117: 1306-1313.

25. Rosenwasser LJ, Klemm DJ, Dresback JK, Inamura H, Mascali JJ, et al. (1995) Promoter polymorphisms in the chromosome 5 gene cluster in asthma and atopy. Clin Exp Allergy 25 Suppl 2: 74-78; discussion 95-76.

26. Basehore MJ, Howard TD, Lange LA, Moore WC, Hawkins GA, et al. (2004) A comprehensive evaluation of IL4 variants in ethnically diverse populations: association of total serum IgE levels and asthma in white subjects. J Allergy Clin Immunol 114: 80-87.

27. Baldini M, Lohman IC, Halonen M, Erickson RP, Holt PG, et al. (1999) A Polymorphism* in the 5' flanking region of the CD14 gene is associated with circulating soluble CD14 levels and with total serum immunoglobulin E. Am J Respir Cell Mol Biol 20: 976-983.

28. Litonjua AA, Belanger K, Celedon JC, Milton DK, Bracken MB, et al. (2005) Polymorphisms in the 5' region of the CD14 gene are associated with eczema in young children. J Allergy Clin Immunol 115: 1056-1062.

29. Walley AJ, Chavanas S, Moffatt MF, Esnouf RM, Ubhi B, et al. (2001) Gene polymorphism in Netherton and common atopic disease. Nat Genet 29: 175-178.

30. Dewar J, Wheatley A, Wilkinson J, Holgate ST, Thomas NS, et al. (1997) Association of the Gln 27 beta 2-adrenoceptor polymorphism and IgE variability in asthmatic families. Chest 111: 78S-79S.

31. Dewar JC, Wilkinson J, Wheatley A, Thomas NS, Doull I, et al. (1997) The glutamine 27 beta2-adrenoceptor polymorphism is associated with elevated IgE levels in asthmatic families. J Allergy Clin Immunol 100: 261-265.

32. Weidinger S, Klopp N, Rummler L, Wagenpfeil S, Novak N, et al. (2005) Association of NOD1 polymorphisms with atopic eczema and related phenotypes. J Allergy Clin Immunol 116: 177-184.

33. Hysi P, Kabesch M, Moffatt MF, Schedel M, Carr D, et al. (2005) NOD1 variation, immunoglobulin E and asthma. Hum Mol Genet 14: 935-941.

34. Kormann MS, Carr D, Klopp N, Illig T, Leupold W, et al. (2005) G-Protein-coupled receptor polymorphisms are associated with asthma in a large German population. Am J Respir Crit Care Med 171: 1358-1362.

35. Malerba G, Lindgren CM, Xumerle L, Kiviluoma P, Trabetti E, et al. (2007) Chromosome 7p linkage and GPR154 gene association in Italian families with allergic asthma. Clin Exp Allergy 37: 83-89.

36. Kruse S, Kuehr J, Moseler M, Kopp MV, Kurz T, et al. (2003) Polymorphisms in the IL 18 gene are associated with specific sensitization to common allergens and allergic rhinitis. J Allergy Clin Immunol 111: 117-122.

37. Imboden M, Nieters A, Bircher AJ, Brutsche M, Becker N, et al. (2006) Cytokine gene polymorphisms and atopic disease in two European cohorts. (ECRHS-Basel and SAPALDIA). Clin Mol Allergy 4: 9.

38. Duetsch G, Illig T, Loesgen S, Rohde K, Klopp N, et al. (2002) STAT6 as an asthma candidate gene: polymorphism-screening, association and haplotype analysis in a Caucasian sib-pair study. Hum Mol Genet 11: 613-621.

39. Weidinger S, Klopp N, Wagenpfeil S, Rummler L, Schedel M, et al. (2004) Association of a STAT 6 haplotype with elevated serum IgE levels in a population based cohort of white adults. J Med Genet 41: 658-663.

40. Schedel M, Carr D, Klopp N, Woitsch B, Illig T, et al. (2004) A signal transducer and activator of transcription 6 haplotype influences the regulation of serum IgE levels. J Allergy Clin Immunol 114: 1100-1105.

41. Poon AH, Laprise C, Lemire M, Montpetit A, Sinnett D, et al. (2004) Association of vitamin D receptor genetic variants with susceptibility to asthma and atopy. Am J Respir Crit Care Med 170: 967-973.

42. Raby BA, Lazarus R, Silverman EK, Lake S, Lange C, et al. (2004) Association of vitamin D receptor gene polymorphisms with childhood and adult asthma. Am J Respir Crit Care Med 170: 1057-1065.

43. Immervoll T, Loesgen S, Dutsch G, Gohlke H, Herbon N, et al. (2001) Fine mapping and single nucleotide polymorphism association results of candidate genes for asthma and related phenotypes. Hum Mutat 18: 327-336.

44. Holla LI, Schuller M, Buckova D, Vacha J (2004) Neuronal nitric oxide synthase gene polymorphism and IgE-mediated allergy in the Central European population. Allergy 59: 548-552.

45. Zhang Y, Leaves NI, Anderson GG, Ponting CP, Broxholme J, et al. (2003) Positional cloning of a quantitative trait locus on chromosome 13q14 that influences immunoglobulin E levels and asthma. Nat Genet 34: 181-186.

46. Kruse S, Japha T, Tedner M, Sparholt SH, Forster J, et al. (1999) The polymorphisms S503P and Q576R in the interleukin-4 receptor alpha gene are associated with atopy and influence the signal transduction. Immunology 96: 365-371.

47. Howard TD, Koppelman GH, Xu J, Zheng SL, Postma DS, et al. (2002) Gene-gene interaction in asthma: IL4RA and IL13 in a Dutch population with asthma. Am J Hum Genet 70: 230-236.

48. Woitsch B, Carr D, Stachel D, Schmid I, Weiland SK, et al. (2004) A comprehensive analysis of interleukin-4 receptor polymorphisms and their association with atopy and IgE regulation in childhood. Int Arch Allergy Immunol 135: 319-324.

49. Kabesch M, Peters W, Carr D, Leupold W, Weiland SK, et al. (2003) Association between polymorphisms in caspase recruitment domain containing protein 15 and allergy in two German populations. J Allergy Clin Immunol 111: 813-817.

50. Weidinger S, Klopp N, Rummler L, Wagenpfeil S, Baurecht HJ, et al. (2005) Association of CARD15 polymorphisms with atopy-related traits in a population-based cohort of Caucasian adults. Clin Exp Allergy 35: 866-872.
